# β-catenin is required for taste bud cell renewal and behavioral taste perception in adult mice

**DOI:** 10.1371/journal.pgen.1006990

**Published:** 2017-08-28

**Authors:** Dany Gaillard, Spencer G. Bowles, Ernesto Salcedo, Mingang Xu, Sarah E. Millar, Linda A. Barlow

**Affiliations:** 1 Department of Cell & Developmental Biology and the Rocky Mountain Taste & Smell Center, University of Colorado Anschutz Medical Campus, Aurora, Colorado, United States of America; 2 Departments of Dermatology and Cell & Developmental Biology, Perelman School of Medicine, University of Pennsylvania, Philadelphia, Pennsylvania, United States of America; The University of North Carolina at Chapel Hill, UNITED STATES

## Abstract

Taste stimuli are transduced by taste buds and transmitted to the brain *via* afferent gustatory fibers. Renewal of taste receptor cells from actively dividing progenitors is finely tuned to maintain taste sensitivity throughout life. We show that conditional β-catenin deletion in mouse taste progenitors leads to rapid depletion of progenitors and *Shh*^+^ precursors, which in turn causes taste bud loss, followed by loss of gustatory nerve fibers. In addition, our data suggest LEF1, TCF7 and Wnt3 are involved in a Wnt pathway regulatory feedback loop that controls taste cell renewal in the circumvallate papilla epithelium. Unexpectedly, taste bud decline is greater in the anterior tongue and palate than in the posterior tongue. Mutant mice with this regional pattern of taste bud loss were unable to discern sweet at any concentration, but could distinguish bitter stimuli, albeit with reduced sensitivity. Our findings are consistent with published reports wherein anterior taste buds have higher sweet sensitivity while posterior taste buds are better tuned to bitter, and suggest β-catenin plays a greater role in renewal of anterior versus posterior taste buds.

## Author summary

By remaining relatively constant throughout adult life, the sense of taste helps keep the body healthy. However, taste perception can be disrupted by various environmental factors, including cancer therapies. Here, we show that Wnt/β-catenin signaling, a pathway known to control normal tissue maintenance and associated with the development of cancers, is required for taste cell renewal and behavioral taste sensitivity in mice. Our findings are significant as they suggest that chemotherapies targeting the Wnt pathway in cancerous tissues may cause taste dysfunction and further diminish the quality of life of patients.

## Introduction

The capacity of the body to discriminate beneficial, nutrient-rich food from harmful compounds is key to survival, and multicellular taste buds in the oral cavity are essential for this discrimination. In mice, taste buds reside primarily in specialized taste papillae on the tongue surface; fungiform papillae (FFP) are distributed in the anterior two thirds of the tongue, each housing a single taste bud, whereas a single large circumvallate papilla (CVP) located posteriorly at the dorsal midline, and foliate papillae (FolP) situated posteriorly and bilaterally, contain several hundred buds each. Additionally, taste buds are present in the soft palate (SP), where they are embedded directly in the epithelium. While taste buds in each of these taste fields can detect all taste stimuli including sweet, sour, salty, bitter and umami/savory [1, 2], the anterior FFP and SP taste buds are more attuned to sweet stimuli, while posterior FolP and CVP taste buds are more sensitive to bitter stimuli [3–10].

Murine taste buds comprise a heterogeneous collection of roughly 60 elongate epithelial cells. Taste cells have functional characteristics of neurons, in that they transduce taste stimuli into electrochemical signals *via* release of neurotransmitter onto sensory nerve fibers [11, 12]. However, taste cells are epithelial, and like skin and intestinal epithelium, are continually and rapidly renewed throughout adult life. Although one report suggests a minor potential contribution of the underlying connective tissue to taste cell renewal [13], it has been clearly demonstrated that taste cells derive mainly from epithelial cytokeratin (Krt) 5^+^/Krt14^+^ progenitor cells situated at the basement membrane outside of taste buds [14, 15]. These basally located progenitors produce post-mitotic daughter cells that can either differentiate as non-taste keratinocytes or enter taste buds and transiently express *Sonic Hedgehog* (*Shh*). *Shh*^+^ basal cells within taste buds [16], also known as type IV cells [17–19], are immediate post-mitotic precursors of each of the three morphological types of elongate taste cells [20], including: the most prevalent glial-like Type I cells that likely act as support cells [21–23]; sweet, bitter and umami Type II taste receptor cells [24–28]; and the least common Type III taste receptor cells that detect sour [29–31] and sodium salt at high concentration [32–35]. While the general lingual epithelium renews every 3–4 days [36–38], taste cells are significantly longer-lived with half-lives estimated at 8 and 22 days, for Type II and III cells, respectively [39].

The Wnt/β-catenin pathway is a powerful regulator of homeostasis and regeneration in a variety of tissues, including hair follicles, skin, and intestine [40–48], and activating mutations in the pathway are key drivers of numerous cancers [49]. Wnt ligands bind to Fzd/LRP co-receptors to activate β-catenin, the downstream effector of canonical Wnt signaling, which complexes with TCF/LEF transcription factors to regulate transcription of target genes [49]. We showed previously that β-catenin is also a key regulator of taste cell renewal. Using Wnt/β-catenin reporter mice, we found Wnt signaling is active in several steps in the taste cell lineage, including taste progenitor cells, *Shh*^+^ precursor cells and subsets of each of the 3 differentiated taste cell types [50, 51]. Stabilization of β-catenin in Krt5^+^ progenitors promotes their proliferation and drives daughter cells to differentiate predominantly as Type I and to a lesser extent Type II taste cells [15]. Conversely, genetic deletion of the Wnt ligand Wnt10a causes decreased taste progenitor proliferation and depletion of taste buds [51].

Here, we show that β-catenin is required for taste bud cell renewal in all oral taste fields, including FFP, SP, CVP and FolP. Conditional β-catenin knock-out in Krt5^+^ progenitors (Loss-of-function, Krt5-β-catenin LOF) reduces both proliferation of progenitors and the number of *Shh*^+^ taste precursor cells, which in turn results in progressive decreases in taste bud number and size. In contrast to gain-of-function studies where taste cell Types I and II are affected, Krt5-β-catenin LOF leads to steady depletion of all three taste cell types. While it is well known that taste buds are maintained by an intact innervation [52–54], we find the gustatory innervation likewise depends upon the continued presence of taste buds. Additionally, we show Wnt pathway transcription factors LEF1 and TCF7 and secreted Wnt3 ligand are specifically downregulated in the CVP epithelium of mutant mice, suggesting a positive Wnt signaling feedback loop regulates maintenance of taste cell renewal. Finally, we show the degree of taste bud loss differs by taste field, where anterior FFP and SP taste buds disappear almost completely and do so more rapidly compared with a slow, steady reduction in posterior CVP and FolP taste buds. Consistent with the higher sweet sensitivity of FFP and SP taste buds versus those of the CVP and FolP more attuned to bitter, we find, using taste behavior assays, that early loss of FFP and SP taste buds in mutants correlates with early abolition of sweet perception, followed by reduced, albeit persistent, behavioral detection of bitter at later times, paralleling the decline of posterior taste buds.

## Results

To explore the role of β-catenin in taste cell homeostasis, we used a doxycycline-inducible genetic system to delete β-catenin in Krt5^+^ progenitor cells and their descendent lineage (Krt5rtTA;tetOCre;Catnb^flox(exon2-6)^: “Krt5-β-catenin LOF”) [15, 55]. To confirm efficiency of β-catenin deletion, we assayed β-catenin protein expression in Krt5-β-catenin LOF mice and controls lacking at least one allele following administration of doxycycline chow for 4 weeks. Control CVP and FFP epithelia, including basal keratinocytes residing along the basement membrane which comprise progenitor cells, were strongly β-catenin immunopositive (S1A and S1B Fig white arrowheads), while in Krt5-β-catenin LOF this population lacked β-catenin immunostaining (S1A’ and S1B’ Fig yellow arrowheads). Non-taste cells are rapidly replaced from Krt5^+^ progenitors (~3–4 days) [14, 36–38, 56], whereas taste bud cells are longer lived, with an average lifespan of 2 weeks, with subsets of taste cells persisting for 6 weeks [39, 57, 58]. In line with this, in Krt5-β-catenin LOF mice induced for 4 weeks, β-catenin was abolished in non-taste epithelial cells localized between taste buds (S1A’ Fig, white arrows), and while reduced within taste buds, β-catenin immunostaining revealed longer lived taste cells that were generated prior to doxycycline induction (S1A’–S1B’ Fig, asterisks).

### Progenitor cells are depleted in the absence of β-catenin

**Fig 1:**
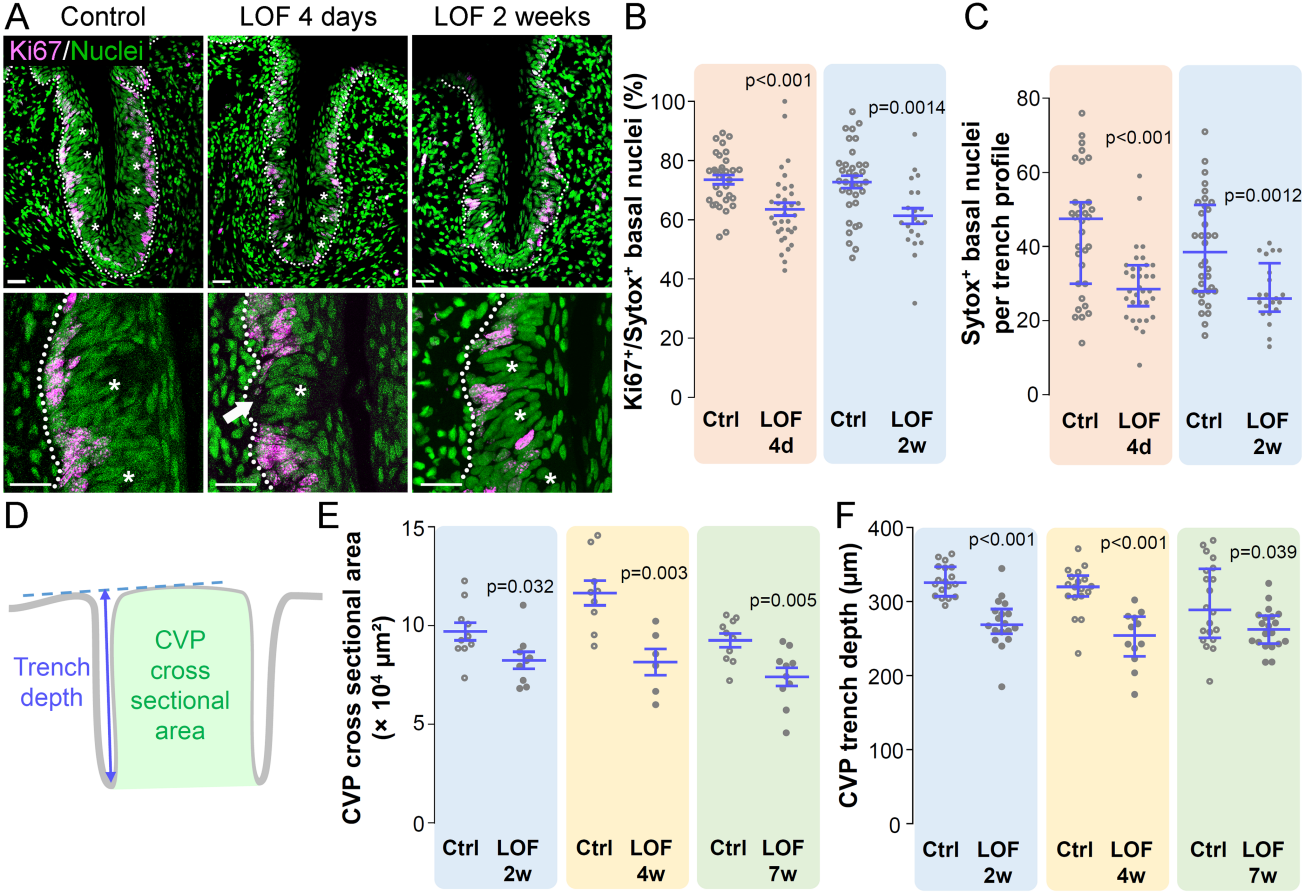
Krt5-β-catenin-LOF results in reduced basal keratinocytes and proliferation, and leads to a smaller CVP. **(A)** Many proliferating Ki67^+^ cells (magenta) are evident at the basement membrane of a control CVP, while in Krt5-β-catenin LOF mutants at 4 days and 2 weeks, fewer Ki67^+^ cells are evident in each CVP trench. Representative images are compressed z-stacks. Nuclei were counterstained with Sytox Green (green). Dotted line delineates the basement membrane. Taste buds are marked with asterisks. Scale bars = 20 μm. **(B)** Progenitor proliferation was significantly decreased in LOF mutants compared to controls at both 4 days and 2 weeks. **(C)** The total number of basal progenitor cells residing along the basement membrane was significantly lower in mutants compared to controls after 4 days and 2 weeks of Krt5-β-catenin LOF (white arrow). CVP size was assessed *via* the cross sectional area between the two trenches (**D**, green area), and CVP trench depth (**D**, blue arrow). By 2 weeks of doxycycline chow, CVP size and trench depth were reduced in mutant mice compared with controls (**E**,**F**). Data are represented as scatter plots (individual symbols), and mean ± SEM (**B**,**E**, blue bars. Student’s t-test) or median with 1^st^ and 3^rd^ quartile (**C**,**F**, blue bars. Mann & Whitney test). Sample sizes: (**B**,**C**) 32 vs 34 CVP trench sections at 4 days and 34 vs 21 CVP trench sections at 2 weeks from 3 control mice vs 3 mutant mice, respectively. **(E)** 6–10 CVP profiles from 3–4 control mice and 3 mutant mice; **(F)** 12–20 CVP trench profiles from 3–4 control mice and 3 mutant mice.

Krt5^+^ lingual progenitors divide and give rise to post-mitotic cells that differentiate into non-taste and taste cells [14, 17–20]. In control CVP, we found ~75% of basal progenitors are Ki67^+^ and thus engaged in the cell cycle (see Methods for details, Fig 1A–1C), comparable to previous reports [15, 59]. In mutant CVP, the proportion of Ki67^+^ progenitor cells was reduced at 4 days, and 2 weeks (Fig 1A and 1B). In addition, Krt5-β-catenin LOF resulted in significantly fewer basally located epithelial cells, quantified via Sytox Green^+^ nuclear counterstain at both 4 days and 2 weeks (See Methods, Fig 1A and 1C, white arrow), indicating reduced proliferation led to depletion of basal keratinocytes, *i*.*e*. taste progenitor cells, in this time frame.

Krt5^+^ progenitor cells are thought to comprise self-renewing stem cells and a population of rapidly replaced, transit amplifying (TA) cells [14, 60]. Fewer basal cells in the trenches of mutant CVP (see above and Fig 1A and 1C) therefore may represent a reduction in stem cells and/or TA cells, which would lead to fewer post-mitotic daughter cells contributing to both taste and non-taste epithelium. Thus, we reasoned the CVP would become progressively smaller in Krt5-β-catenin LOF mutants. Consistent with our hypothesis, both CVP cross sectional area (Fig 1D and 1E) and the depth of taste bud-bearing epithelial trenches (Fig 1D and 1F) were significantly reduced in mutant compared to control mice after 2, 4 and 7 weeks on doxycycline chow. Noticeably, decline in CVP morphometric does not increase at longer time points, suggesting other factors may compensate for Krt5-β-catenin LOF. Indeed, longer time point measurements will be helpful in determining β-catenin’s role in maintenance of taste cell homeostasis.

### *Shh*^+^ post-mitotic taste precursor cells are reduced in Krt5-β-catenin LOF mice

**Fig 2:**
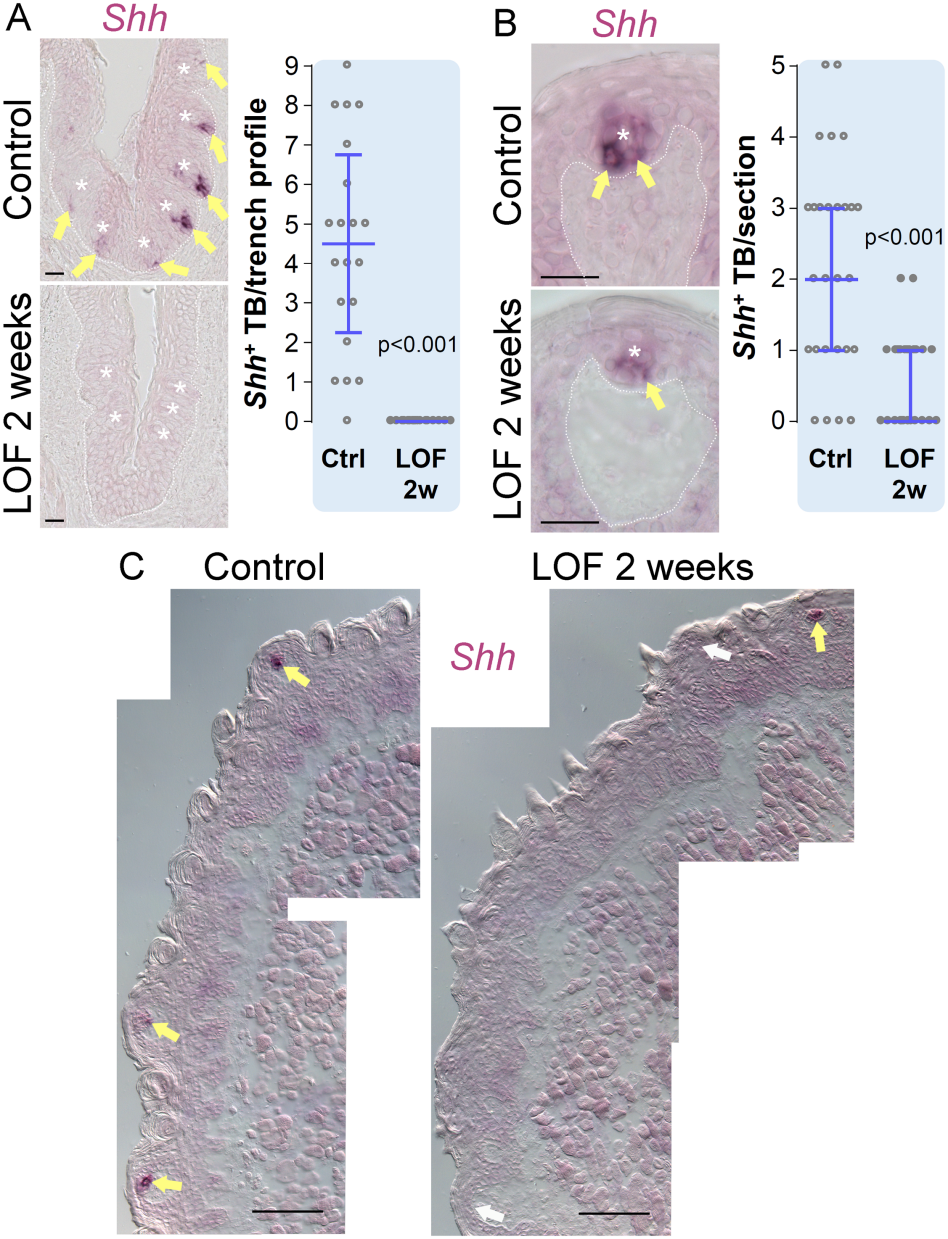
Beta-catenin deletion in Krt5^+^ progenitors reduces *Shh* expressing taste precursor cells in CVP and FFP taste buds. In control mice, *Shh* is expressed in taste precursors that give rise to all three cell types (**A**,**B**,**C**, yellow arrows). In the CVP, *Shh* expression is abolished in mutants after 2 weeks on doxycycline chow (**A**), while the number of *Shh*^+^ taste buds is greatly reduced in mutant FFP (**B**,**C**). Dotted line delineates the basement membrane. Taste buds are marked with asterisks. *Shh*^-^ FFPs are marked with white arrows. TB: taste bud. Data are represented as scatter plots (individual symbols), and median with 1^st^ and 3^rd^ quartile (blue bars. Mann & Whitney test). **(A)** 18–20 CVP trench profiles. **(B)** 27 FFP profiles. N = 3 control mice and 3 mutant mice. Scale bars in **A**, **B** = 20 μm, **C** = 100 μm.

Taste progenitors give rise to post-mitotic *Shh*^+^ precursor cells that differentiate into Type I, II and III taste cells. Therefore, we predicted a smaller progenitor population with reduced proliferation would lead to fewer *Shh*^+^ precursor cells in the taste buds of mutant mice. In controls, *Shh*^+^ precursor cells are normally found in the basal compartment of taste buds (Fig 2A, 2B and 2C yellow arrows). However, the number of FFPs housing *Shh*^+^ cells was significantly reduced in mutants examined at 2 weeks (Fig 2B and 2C); further, *Shh*^+^ cells were completely absent in CVP taste buds of mutants at this time point (Fig 2A). These data suggest absence of β-catenin leads to a failed production of new taste precursor cells.

### Taste bud number and size are reduced by deletion of β-catenin in Krt5^+^ progenitors

**Fig 3:**
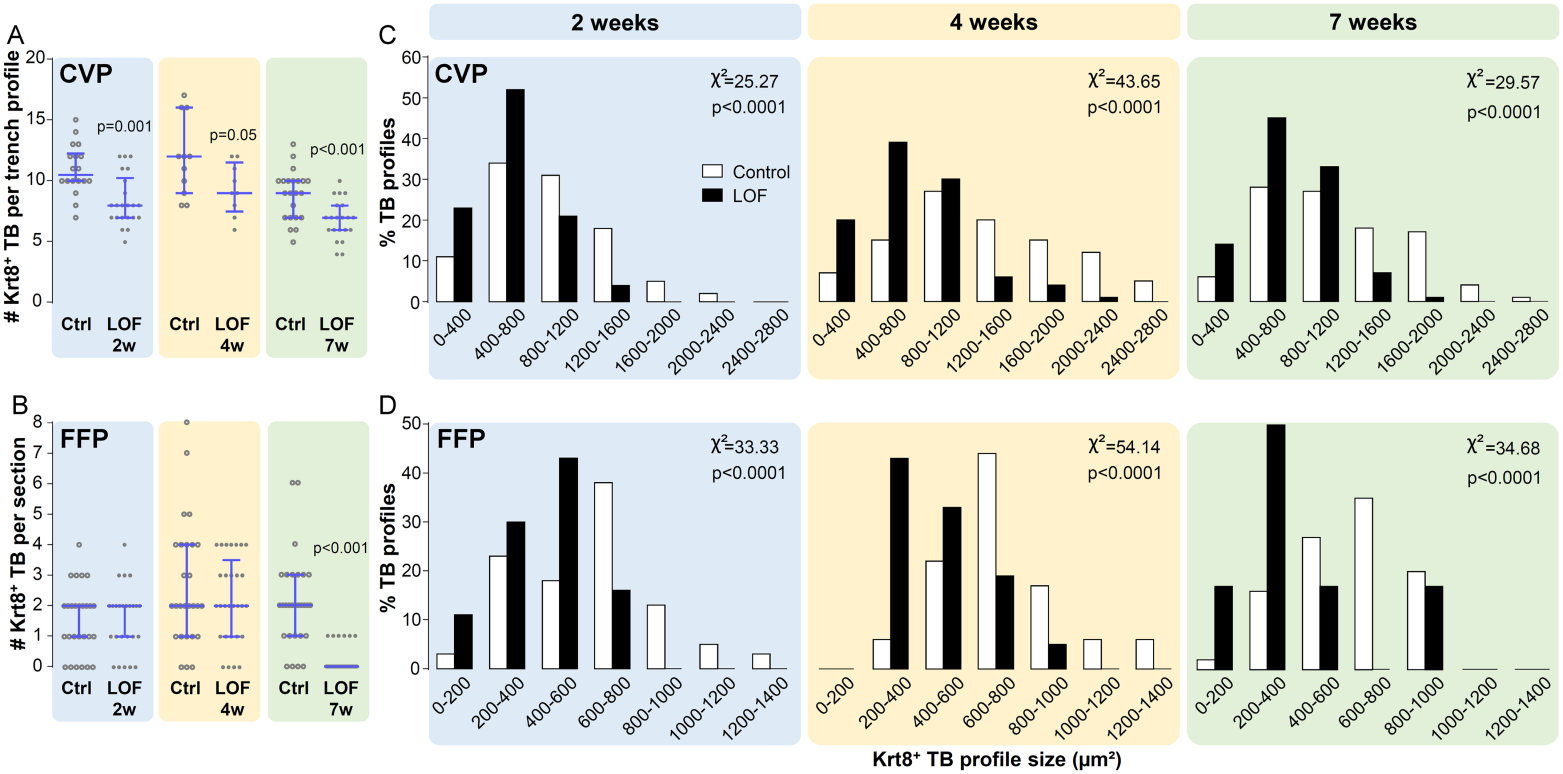
Taste bud number and size are reduced by deletion of β-catenin in Krt5^+^ progenitors. **(A)** The number of Krt8^+^ taste buds was significantly reduced in the CVP of mutants by 2 weeks on doxycycline chow, and this reduction remained relatively constant at 4 and 7 weeks. **(B)** The number of Krt8^+^ FFP taste buds in the anterior tongue did not differ between mutants and controls at 2 and 4 weeks of β-catenin deletion, but Krt8^+^ taste buds were virtually absent in mutant tongues at 7 weeks; only 6 FFP taste buds were observed in 5 mutant animals, compared to 51 in 4 controls. Both the CVP (**C**) and FFP (**D**) of mutant mice housed smaller taste buds than those of controls after 2, 4 and 7 weeks of doxycycline chow. TB: taste bud. Data are represented as scatter plots (individual symbols), and median with 1^st^ and 3^rd^ quartile (**A**,**B** blue bars. Mann & Whitney test), or taste bud size distribution (**C**,**D** Two-sample chi-square for trend). Sample sizes: **(A)** 9–22 CVP trench profiles from 3–4 control mice and 3 mutant mice per time point; **(B)** 25–59 anterior tongue sections from 3–4 control mice and 3–5 mutant mice per time point; **(C)** 84–198 CVP taste bud profiles from 3–4 control mice and 3 mutant mice per time point; **(D)** 6–51 FFP taste bud profiles from 4 control mice and 5 mutant mice per time point.

If β-catenin is required for taste bud cell renewal, we reasoned β-catenin deletion in Krt5^+^ progenitors would lead to steady run-down of taste buds, as old taste cells are lost *via* natural attrition and insufficiently replaced from a depleted progenitor population. To address this prediction, we first assessed taste bud number and size *via* immunostaining for Krt8, a general marker of differentiated taste cells [61, 62]. In the CVP, taste bud number was significantly reduced in mutants after 2 weeks of doxycycline, and continued to decline to roughly half of controls over the following weeks (Fig 3A). By contrast, FFP taste bud number did not differ between mutants and controls at 2 and 4 weeks, but surprisingly taste buds were virtually absent in mutant tongues at 7 weeks (Fig 3B).

In tissue sections, taste bud size can be estimated by outlining the area occupied by Krt8^+^ immunostaining; in controls, Krt8^+^ taste bud profile area ranges from 400–2800 μm^2^ in CVP (white bars in Fig 3C) and 200–1400 μm^2^ in FFP (white bars in Fig 3D). As soon as 2 weeks, mutant taste buds were significantly smaller in CVP and FFP (black bars in Fig 3C and 3D, respectively), and mutant taste buds were progressively smaller with prolonged doxycycline chow feeding; even the few FFP taste buds remaining at 7 weeks were significantly reduced in size (black bars in Fig 3D far right histogram).

The size of taste buds in foliate papillae (FolP) and soft palate (SP) were similarly affected by Krt5-β-catenin LOF. After 7 weeks, mutant FolP (S2A–S2C Fig) and SP (S2D–S2F Fig) housed fewer and smaller taste buds compared with controls. Interestingly, the mutant phenotype of posteriorly located FolP resembled that of the CVP, as a substantial number of smaller taste buds was still evident, while in the SP, taste buds were almost completely absent, reminiscent of the impact of β-catenin deletion on FFP.

### Beta-catenin is essential for the renewal of type I, II and III taste cells

**Fig 4:**
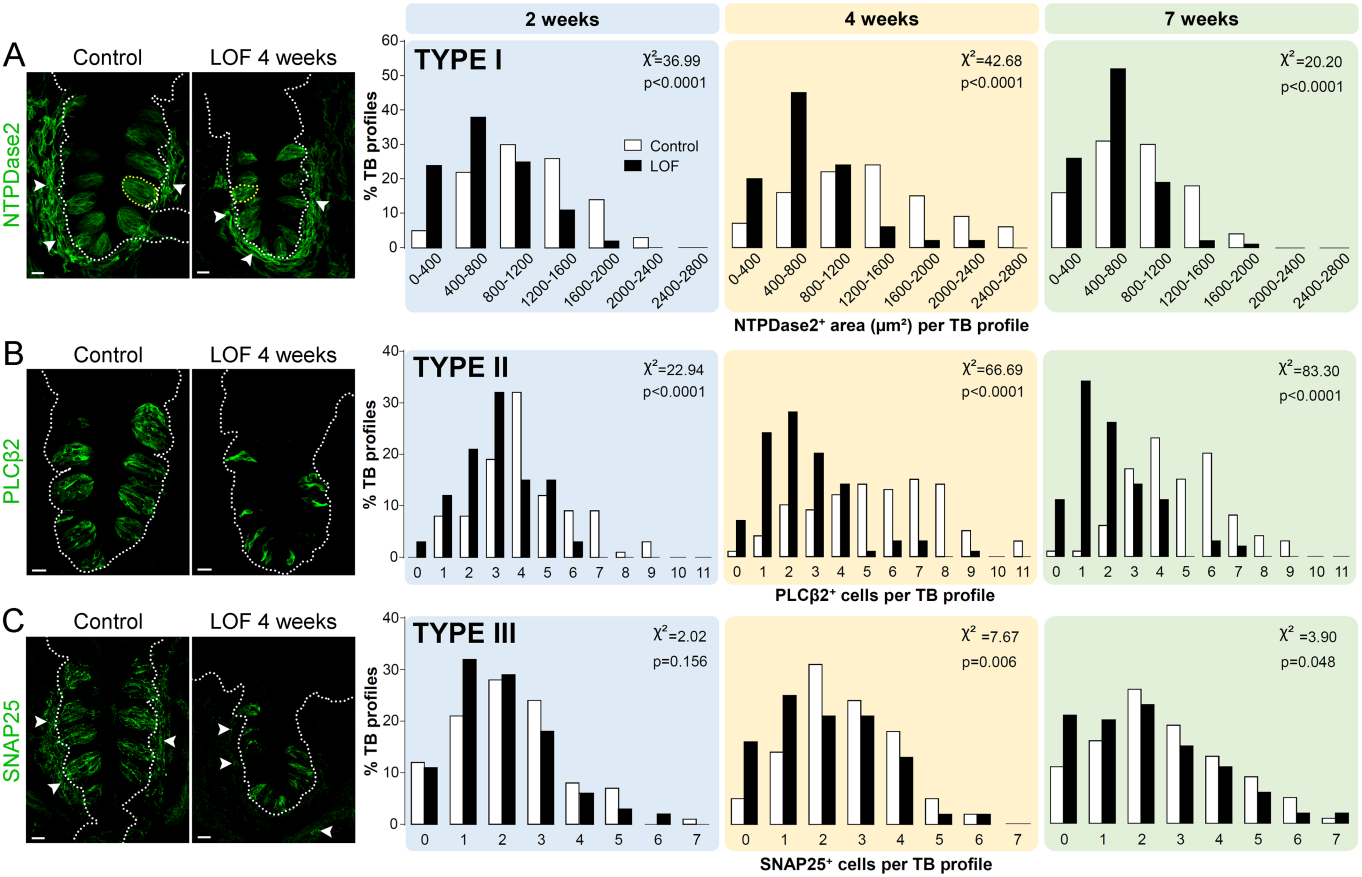
Krt5-β-catenin LOF reduces all 3 taste cell types in the CVP. **(A)** NTPDase2 (green) marks the membranes of Type I glial-like taste cells, as well as a subset of mesenchymal cells adjacent to the CVP epithelium (white arrowheads). The area of taste buds (selected by outlining Krt8^+^ immunostaining) occupied by NTPDase2^+^ signal within the CVP epithelium is a proxy for Type I cell prevalence (yellow dotted circles; and see Methods), and NTPDase2^+^ area per taste bud profile become progressively smaller in mutants (black bars, yellow dotted circle) compared to controls (white bars, yellow dotted circle). **(B)** PLCβ2 (green) marks the cytoplasm of Type II taste cells, and individual cells are easily counted. In mutants, the number of PLCβ2^+^ Type II cells per taste bud is significantly reduced in mutant CVP at all time points. **(C)** SNAP25 (green) is expressed by Type III cells, and by nerve fibers innervating lingual epithelium (white arrowheads). Type III cells per taste bud in mutants did not differ from controls at 2 weeks, and were only mildly reduced in the CVP of Krt5-β-catenin LOF mice at 4 and 7 weeks. TB: taste bud. Taste cell type data are presented as the number of taste buds falling into each bin along the X axis (NTPdase2^+^ area per Krt8^+^ bud profile in **A**, PLCβ2^+^ cell number per Krt8^+^ bud profile in **B**, and SNAP25^+^ cell number per Krt8^+^ bud profile in **C**). For all histogram panels, significance was determined by a Two-sample chi-square for trend. Sample sizes: **(A)** 82–227 CVP taste bud profiles; **(B)** 34–216 CVP taste bud profiles; **(C)** 118–308 CVP taste bud profiles, from 3–6 control mice and 3–5 mutant mice at each time point. Representative images are compressed z-stacks. Dotted lines delineate basement membrane. Scale bars = 20 μm.

In mice, taste buds are collections of ~60 Type I, II and III taste cells. Activation of β-catenin in Krt5^+^ progenitors drives daughter cells to become primarily Type I, and, to a lesser extent, Type II cells [15]. Hence, we next addressed whether the reduction in taste bud size in Krt5-β-catenin LOF mice was due to a decrease in one or more taste cell types. Type I cells are thought to function as glial-like cells within taste buds, and have extensive cellular processes that tightly wrap other taste cells [22, 23]. The only marker of Type I cells to date, NTPDase2, is membrane-associated [21], and thus NTPDase2 immunostaining does not allow individual Type I cells to be tallied in tissue sections (see [20], their Figs 4, 5). Instead, using the area occupied by NTPDase2 immunofluorescence per taste bud profile (selected *via* outlining Krt8^+^ immunostaining) to estimate relative Type I cell populations, we found NTPDase2^+^ area was significantly smaller in CVP and FFP taste buds of mutant mice at all time points (Fig 4A, S3A Fig).

Type II cells transduce sweet, bitter and umami tastants *via* a common intracellular transduction cascade that includes PLCβ2 [63]. Type II cells are thus readily quantified *via* cytosolic PLCβ2 immunoreactivity. Consistent with a requirement for β-catenin, the number of Type II cells per CVP taste bud was significantly reduced by 2 weeks (~3 PLCβ2^+^ cells per mutant bud compared to 4 cells per control bud); by 7 weeks, most mutant CVP taste buds housed only 1–2 Type II cells (Fig 4B). Similarly, mutant FFP taste buds possessed fewer PLCβ2^+^ Type II cells by 2 weeks of doxycycline induction, and at all subsequent times (S3B Fig).

Type III taste cells transduce sour stimuli [29, 30] and sodium salt [32, 33], and can be identified *via* SNAP25 immunostaining [64]. Mutant and control CVPs had comparable numbers of SNAP25^+^ Type III cells per bud at 2 weeks, while at 4 and 7 weeks, mutant buds housed slightly, albeit significantly, fewer Type III cells than those of controls (Fig 4C). A slower decline in Type III cells in mutant taste buds is consistent with the fact that Type III taste cells persist up to 45 days in control buds, and are replaced less often than the shorter lived Type I and II taste cells [20, 39, 58]. In FFP, reduction in SNAP25^+^ Type III cells per bud was evident at 4 weeks, and Type III cells were almost totally absent at 7 weeks (S3C Fig), consistent with the broad loss of FFP taste buds.

### Taste bud innervation is affected by loss of β-catenin in taste epithelium

**Fig 5:**
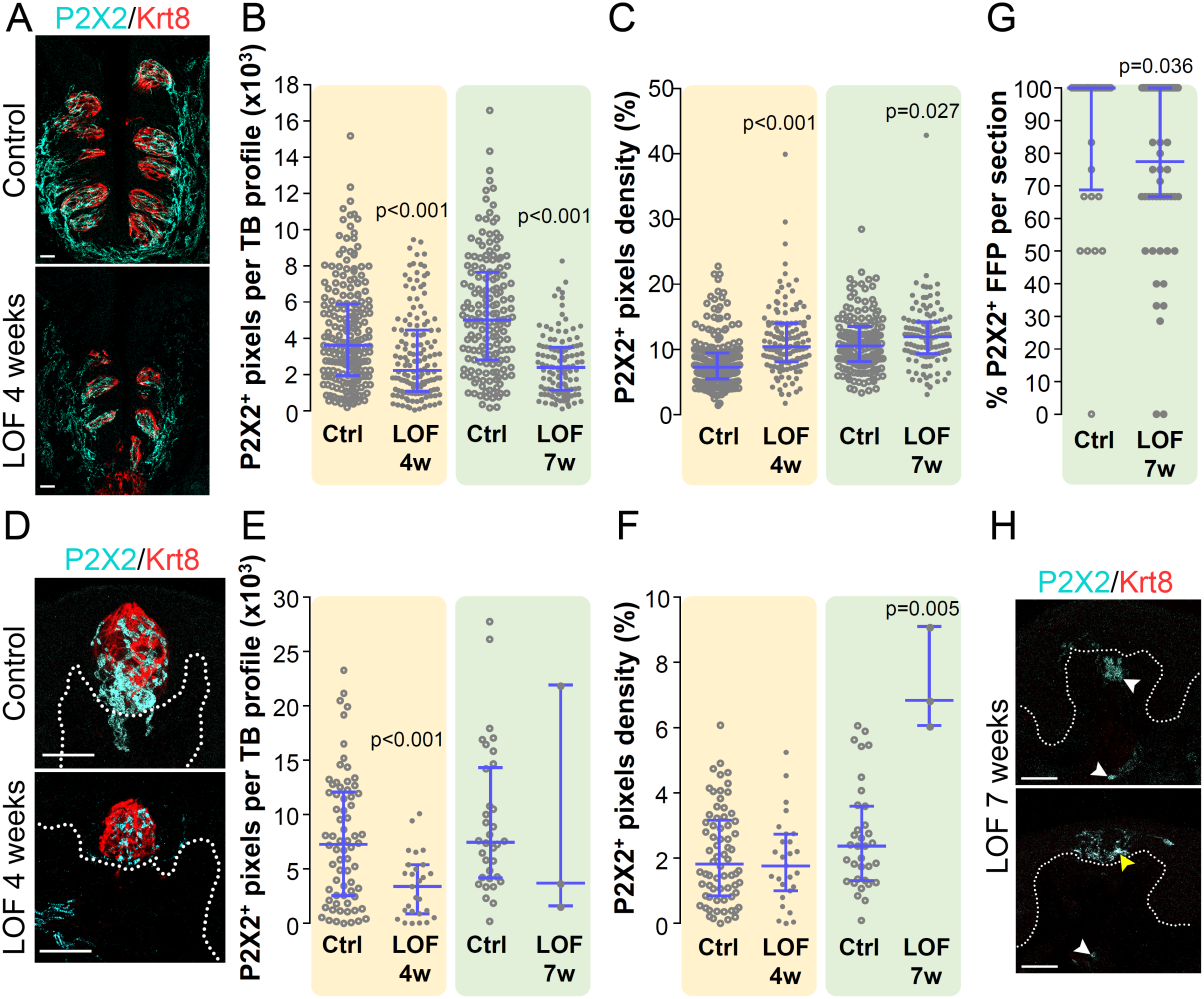
Gustatory innervation is reduced following loss of taste buds in Krt5-β-catenin LOF mice. P2X2 is expressed by gustatory fibers innervating taste buds. In mutants, CVP taste buds had fewer P2X2^+^ pixels than controls at 4 and 7 weeks of doxycycline chow (**A**,**B**); however, because CVP taste buds become smaller in mutants, the density of P2X2^+^ pixels per bud increased (**C**). Total P2X2^+^ pixels per FFP taste bud were reduced in mutant mice fed doxycycline for 4 weeks (**D**,**E**), while P2X2^+^ innervation of the few remaining taste buds at 7 weeks was highly variable (**E**). P2X2^+^ pixel density in mutant FFP did not differ from controls at 4 weeks, despite the smaller size of mutant taste buds at this time (see Fig 3D), but was significantly greater in the few remaining taste buds in mutants at 7 weeks (**F**). In the absence of Krt8^+^ taste buds, empty FFP are identified via P2X2^+^ fibers. Compared to controls, fewer P2X2^+^ FFP were present in mutant mice fed doxycycline for 7 weeks (**G**,**H**); of these, P2X2^+^ fibers were evident in the mesenchymal core of FFP (**H**, white arrowheads), and were seldom observed in the epithelium (**H**, yellow arrowhead). Data are represented as scatter plots (individual symbols), and median with 1^st^ and 3^rd^ quartile (blue bars. Mann & Whitney test). Sample sizes: **(B**,**C)** 125–215 CVP taste bud profiles from 3–4 control mice and 3–4 mutant mice per time point; **(E**,**F)** 34–70 FFP taste bud profiles from 3–4 control mice and 3–27 FFP taste bud profiles from 3 mutant mice per time point; **(G)** 32–54 FFP profiles from 3–4 control mice and 3–4 mutant mice per time point. Note: low numbers of taste buds observed represent those measured at 7 weeks, when few FFP taste buds remained in all animals. **(A**,**D**,**H)** Representative images are compressed z-stacks. Dotted lines delineate the basement membrane. Scale bars = 20 μm.

Taste cells transmit gustatory signals to the brain *via* taste sensory afferents, and maintenance of mammalian taste buds is highly dependent upon this innervation [52–54]. However, the degree to which the gustatory innervation is maintained by taste buds has not been assessed. As a large subset of gustatory sensory neurons express the purinergic receptor, P2X2 [65, 66], we compared the extent of P2X2^+^ nerve fibers in mutant versus control taste buds (See Methods). The total number of P2X2^+^ pixels in mutant taste buds was significantly reduced in both the CVP and FFP at 4 and 7 weeks compared to controls (Fig 5A, 5B, 5D and 5E). However, because CVP taste buds are smaller in Krt5-β-catenin LOF mice, the density of P2X2^+^ innervation was significantly higher in mutant CVP buds compared to controls (Fig 5C). Similarly, despite reduction in overall P2X2^+^ fibers in the smaller taste buds of mutant FFP, the density of P2X2^+^ innervation remained comparable to controls at 4 weeks and was higher at 7 weeks in the few remaining, albeit significantly smaller FFP buds (Fig 5E and 5F). These data suggest intact taste buds maintain taste innervation, and as taste buds are diminished in size and cell number, their innervation likewise is reduced. Consistent with the idea that innervation loss follows taste bud loss, we found that P2X2^+^ pixels were still present in large numbers of FFP in mutants, where most Krt8^+^ taste buds had been lost yet some gustatory innervation remained (Fig 5G and 5H). In these FFP, P2X2^+^ pixels were observed in the taste papilla epithelium and mesenchyme (Fig 5H, yellow and white arrowheads, respectively).

### LEF1, TCF7 and Wnt3 are downregulated in CVP epithelium in the absence of β-catenin

**Fig 6:**
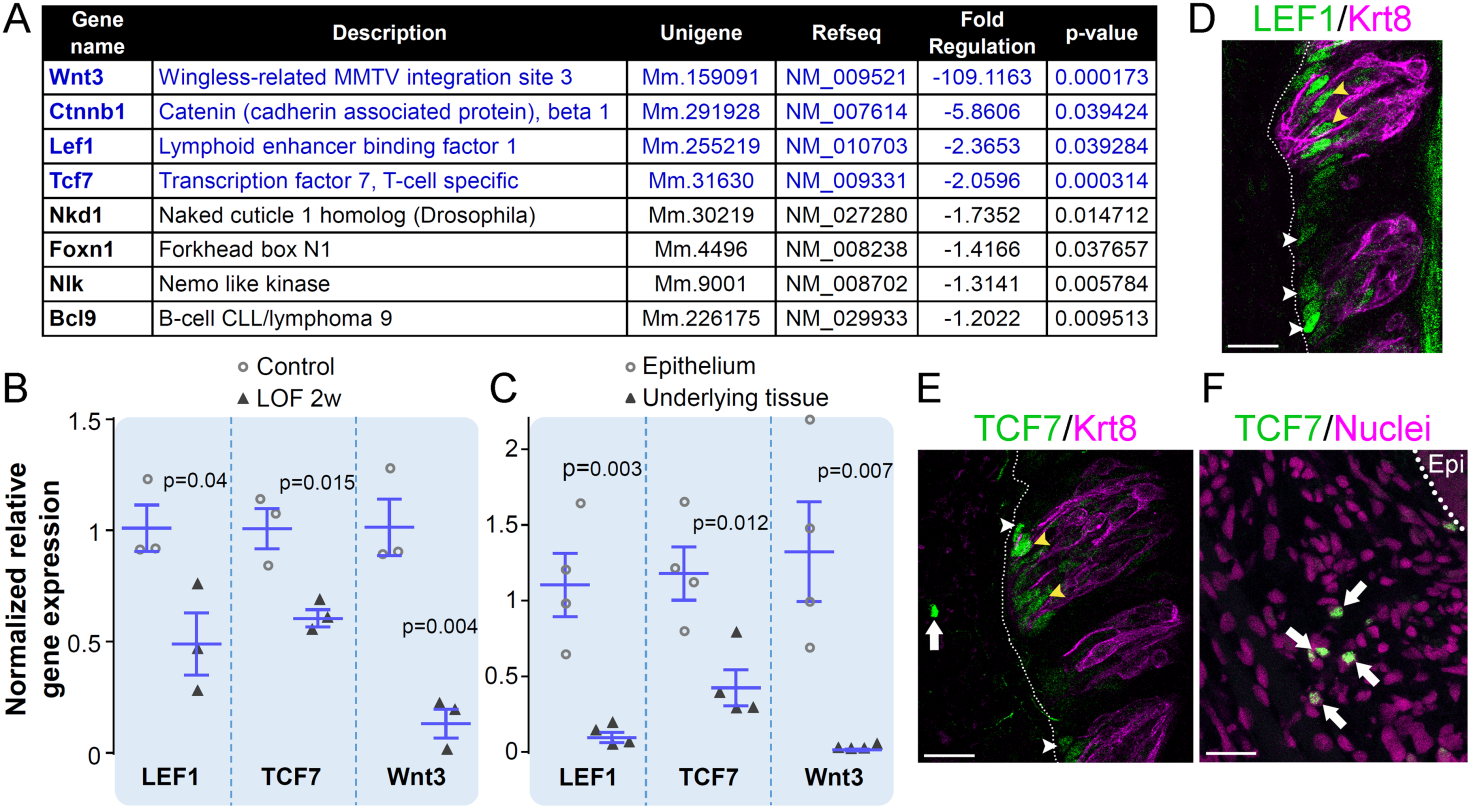
LEF1, TCF7 and Wnt3 are downregulated in Krt5-β-catenin LOF CVP. To identify Wnt pathway genes regulated in the absence of β-catenin, a Wnt pathway RT^2^ profiler PCR assay was run on RNAs extracted from whole CVP of Krt5-β-catenin LOF mice and their control counterparts (see Methods). As expected, β-catenin expression was downregulated 6 fold in Krt5-β-catenin LOF mice compared with controls (**A**). Seven other genes were significantly regulated; all being downregulated (**A**) and attention was focused on genes which expression changed more than 2 fold, *i*.*e*. LEF1, TCF7 and Wnt3 (**A**, genes in blue). Regulation of LEF1, TCF7 and Wnt3 expression was confirmed by qRT-PCR (**B**). qRT-PCR analysis of epithelium versus underlying tissue (mesenchyme and Von Ebner’s glands) of wild-type CVPs revealed that LEF1 and Wnt3 are specifically expressed in the epithelium whereas expression of TCF7 is predominant in the epithelium and lower in the underlying tissue (**C**). Immunolabelling of LEF1 and TCF7 is localized in perigemmal basal cell, which include progenitors (**D**,**E**, white arrowheads) and within taste buds (**D**,**E**, yellow arrowheads). In addition, TCF7 signal was observed in the mesenchyme (**E**,**F**, white arrows) supporting the qRT-PCR data. Dotted line delineates the basement membrane; Epi: CVP trench epithelium. N = 3 mice per group, except **C** (n = 4). Data are represented as scatter plots (individual symbols), and mean ± SEM (blue bars. Student’s t-test). Representative images are compressed z-stacks. Dotted lines delineate the basement membrane. Nuclei were counterstained with DRAQ5. Scale bars = 20 μm.

Beta-catenin is essential for the transcription of Wnt target genes, including Wnt pathway components [49]. To identify Wnt pathway genes regulated by β-catenin in mouse CVP, we compared gene expression between controls and Krt5-β-catenin LOF CVP at 2 weeks using a Wnt pathway profiler PCR assay (see Methods). Eight genes were significantly downregulated in Krt5-β-catenin LOF CVPs (Fig 6A; Complete gene list is presented in S1 Table). As expected, β-catenin expression was reduced by 6 fold. In addition, Nkd1, Foxn1, Nlk and Bcl9 expression was slightly downregulated (< 2 fold, Fig 6A, genes in black). Interestingly, Wnt3, LEF1 and TCF7 RNA levels were highly reduced (> 2 fold, Fig 6A, genes in blue), and these reductions in mutants were confirmed by qRT-PCR (Fig 6B). We next sought to determine in which CVP tissue(s) these three genes are expressed. The CVP of wild type mice were harvested and epithelium separated from underlying mesenchyme and von Ebner’s glands [67–69]. We found LEF1 and Wnt3 expression is restricted to the CVP epithelium, whereas TCF7 is expressed in both tissue compartments, albeit at higher levels in the epithelium (Fig 6C). LEF and TCF proteins complex with β-catenin to bind to LEF/TCF response elements and regulate the transcription of target genes [49]. Thus, we explored specifically which general cell types within the CVP epithelium express LEF1 and TCF7 in wild type mice. Both LEF1 and TCF7 proteins were expressed in the perigemmal compartment where progenitors are normally found (Fig 6D and 6E, white arrowheads), as well as by cells within taste buds, predominantly in the basal compartment where post-mitotic precursor cells are located (Fig 6D and 6E, yellow arrowheads). Expression of TCF7 was also observed in the mesenchyme (Fig 6E and 6F, white arrow), specifically as TCF7-bright cells with small round nuclei, the latter suggestive of lymphocytes [70–72]. Altogether, these data suggest Wnt3, LEF1 and TCF7 function primarily in the taste epithelium to promote taste cell renewal.

### Behavioral discrimination of taste stimuli is reduced in Krt5-β-catenin LOF mice

Taste buds and their innervation are progressively diminished in Krt5-β-catenin LOF mice, suggesting the ability of mice to discern taste stimuli might also be reduced. This hypothesis was further supported by: (1) Our observation that mutant mice fed chow *ad libitum* experienced significant weight loss by 7 weeks (S4A Fig); and (2) in a 48 hour two-bottle taste preference assay (see Methods and [73]), control mice prefer the sweet taste of 3 mM saccharine over water, but this preference is reduced in mutant mice fed doxycycline chow for 4 weeks (S4B Fig).

**Fig 7:**
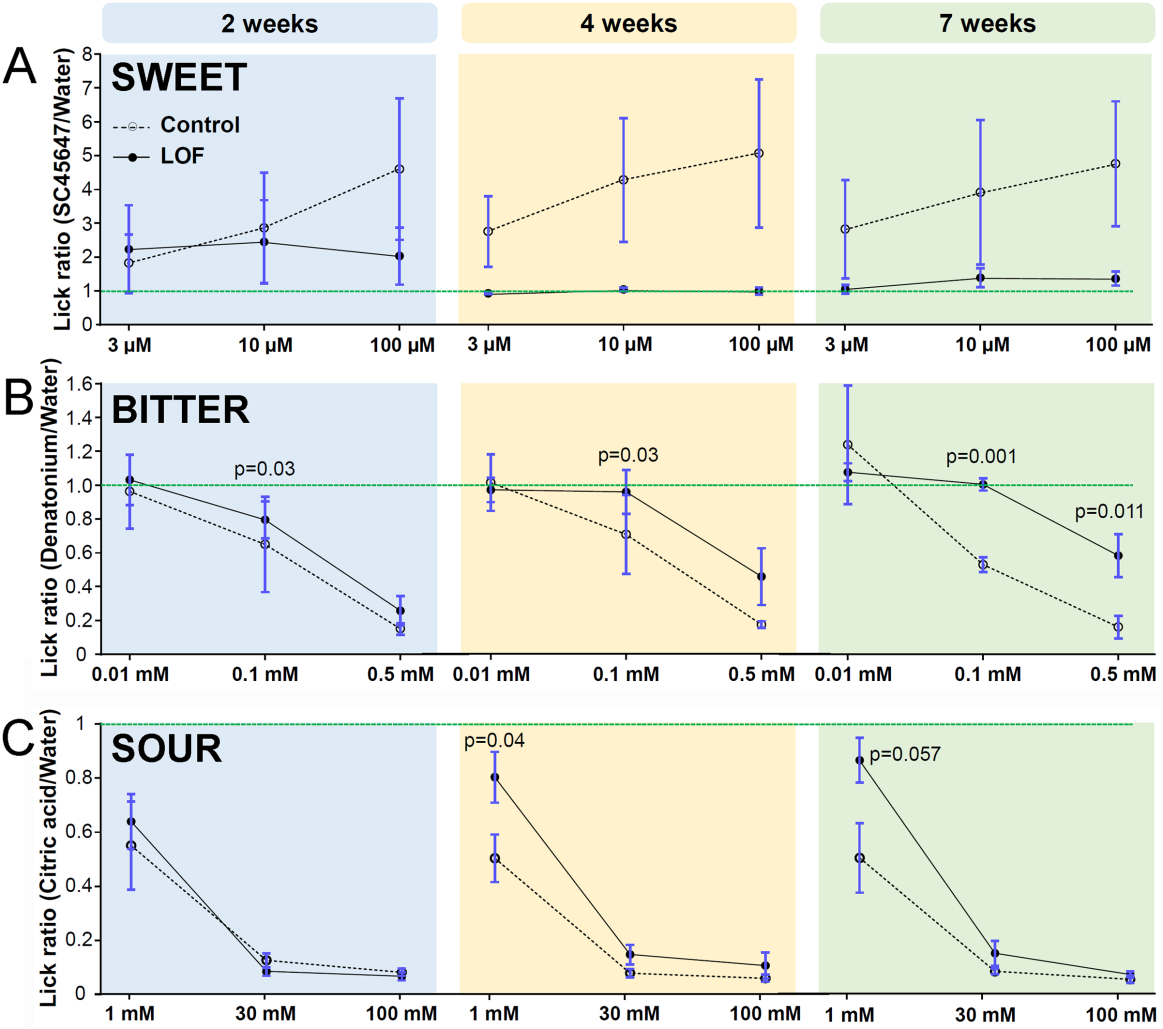
β-catenin deletion in Krt5^+^ progenitors causes early loss of behavioral discrimination of sweet, followed by reduced sensitivity to bitter. Taste sensitivity of control and mutant mice was assessed using brief-access lickometers. By 4 weeks, mutant mice could not distinguish the artificial sweetener SC45647 from water (**A**). (**B**) Detection of the bitter tastant, denatonium, was slightly impaired in mutants at 2 and 4 weeks; by 7 weeks, LOF mice displayed highly reduced sensitivity to bitter. Detection of the lowest concentration of citric acid tested was slightly reduced in mutant mice at 4 and 7 weeks (**C**). The lick ratio is the average number of licks triggered by a tastant stimulus divided by the average number of licks triggered by water; green dash line marks a lick ratio of 1, *i*.*e*. absence of perception of the tastant compared to water. Data are represented as mean ± SEM; Mann & Whitney test used for all panels. N = 8–15 control mice and 6–13 mutant mice per time point.

Because taste perception during the 2 day long two-bottle test can be influenced by post-ingestive signals mediated by the intestine [74], taste behavior of control and mutant mice was tested at progressive time points in a brief-access paradigm using Davis-Rig lickometers (see Methods) [73, 75]. While control mice highly preferred the artificial sweetener SC45647 at 3, 10 and 100 μM over water, mutant mice could not discriminate any concentration of SC45647 from water after 4 and 7 weeks on doxycycline (Fig 7A); a result consistent with the reduced sensitivity to saccharine observed in the two-bottle test above. However, this result was not significant, due to the high variability in the response of control mice.

By contrast, discrimination of a bitter tastant, denatonium benzoate (0.01, 0.1 and 0.5 mM), was less affected in mutants at all time points examined (Fig 7B). While all mice could detect denatonium at the highest concentration (0.5 mM) at all time points, mutant mice showed reduced sensitivity and were unable to distinguish 0.1 mM denatonium from water at 4 and 7 weeks.

Finally, we tested the ability of mice to detect sour stimuli. The taste of citric acid is aversive to mice, and both control and mutant mice showed normal discrimination of sour at 2 weeks (Fig 7C). However, mutant mice did not avoid 1 mM citric acid (the lowest concentration tested) at 4 weeks and tended not to avoid it at 7 weeks (p = 0.057), while controls found 1 mM citric acid aversive. Higher concentrations of citric acid were comparably aversive for mutant and control mice at all times post-induction.

## Discussion

Here, using conditional deletion of β-catenin in Krt5^+^ basal keratinocytes of the tongue epithelium, we show that epithelial β-catenin is required for taste cell renewal and behavioral taste perception. Specifically, progenitor proliferation is reduced, resulting in fewer *Shh*^+^ taste precursor cells and smaller and fewer taste buds; loss of taste buds leads secondarily to loss of gustatory innervation. The impact of β-catenin loss is comparable across taste cell types, as Type I, II and III taste cells are all progressively reduced. In addition, we show that β-catenin is required for expression of components of the Wnt pathway, including the transcription factors LEF1 and TCF7, and Wnt3 ligand that together likely function in CVP epithelium to promote taste cell renewal. Unexpectedly, we found taste bud reduction was accelerated and more extreme in FFP of the anterior tongue and the SP compared to the posterior CVP and FolP. As FFP and SP taste buds are more attuned to sweet tastes, and CVP and FolP to bitter stimuli, the pattern of taste bud loss results in an early loss in the ability of mice to detect sweet, with a reduced, albeit persistent, bitter sensitivity.

### β-catenin is required for maintenance of taste progenitors and continual production of taste bud cells

Wnt/β-catenin signaling is required for homeostasis of a variety of adult tissues, including epidermis and intestinal epithelium and as we show here, for taste bud renewal. In each of these epithelia, Wnt pathway function is required cell autonomously to maintain proliferating progenitors, such that loss of pathway activity leads to depletion of the resident stem population [40–46]. Nuclear β-catenin complexes with LEF and TCF proteins to bind to specific LEF/TCF response elements and activate transcription of target genes [49]. In addition to functioning as a transcriptional coactivator downstream of Wnt ligand signaling, β-catenin also participates in cell adhesion as a key component of adherens junctions [76–78]. Thus, long term deletion of β-catenin is posited to deplete both nuclear and membrane associated pools, and ultimately abolish adhesive contacts of basal progenitors [79–81] (although see [82]).

While activation of β-catenin in progenitors biases daughter cells to differentiate primarily as Type I glial-like cells [15], deletion of β-catenin impacts generation of all 3 taste cell types. All taste cells are continually renewed, as aged cells undergo apoptosis [83, 84] and are replaced by the entry of newly differentiated taste cells generated by Krt5^+^ progenitors. The more rapidly replaced Type I and II cells are reduced in mutant CVP and FFP within 2 weeks of induction, while the decline in the slower renewing Type III cells is more gradual. Taste cell longevity, as stated in most textbooks, is an average of 10–14 days [57, 85], yet the variance in taste cell lifespan is much larger, ranging from 3 days up to 45 days for a subset of Type III cells [39, 58]. Thus, the lesser impact of β-catenin deletion on Type III cells likely reflects their longer lifespan. Although Type I cells are presumed the shortest lived taste cell type [39, 58], they do not seem to be lost faster than Type II and III cells in Krt5-β-catenin LOF mice (see Fig 4 and S3 Fig). One explanation is that Type I cells may comprise subpopulations with heterogeneous lifespans, with some Type I cells living much longer than others as suggested previously from birthdating studies [39]. Alternatively, in the absence of β-catenin, Type I cell morphology and/or expression of NTPDase2 may be altered such that the relationship between taste bud area occupied by NTPDase2^+^ signal no longer corresponds with Type I cell numbers.

Although all taste bud cells are reduced by loss of β-catenin in Krt5^+^ progenitors, we cannot rule out that loss of β-catenin inherited from Krt5^+^ progenitors may also influence taste cell fate. In control mice, a subset of each of the 3 cell types is Wnt-responsive as assessed by Wnt reporter alleles [50, 51] and taste bud cells broadly express Fzd1 and Fzd3 [86], indicating canonical Wnt signaling may function in post-mitotic taste cells. Additionally, β-catenin gain-of-function promotes Type I cell and sparse Type II cell differentiation, but does not alter Type III cell formation [15]. These findings led us to hypothesize that high levels of β-catenin are required for differentiation of Type I and II cells, with low levels permissive for Type III cell differentiation. This model is consistent with reports of different levels of Wnt/β-catenin signaling regulating cell fate in other tissues, including the intestine, hair follicles and the skin [42, 87, 88]. Additionally, β-catenin function within different cell types may reflect interaction with different transcription factors in a cell-type specific manner, as is the case in the non-taste epithelium. In lingual epithelial progenitors, β-catenin complexes with LEF1, while in differentiated lingual keratinocytes, β-catenin interacts with TCF4 [51]. Thus, both transcriptional partners and β-catenin levels may play a role in the different functions of β-catenin in taste cell homeostasis.

### β-catenin regulates expression of LEF1, TCF7 and Wnt3 in taste epithelium

In the absence of β-catenin in lingual progenitors, we find downregulated expression of LEF1 and TCF7, which are expressed in both progenitor cells, those perigemmal, *i*.*e*. adjacent to, and intragemmal cells within CVP taste buds. Both LEF1 and TCF7 sequences have functional β-catenin/TCF/LEF DNA response elements [89–92], and thus downregulation of LEF1 and TCF7 in the CVP of Krt5-β-catenin LOF mice suggests LEF1 and TCF7 are β-catenin target genes involved in taste cell renewal. While LEF1 is considered primarily a transcriptional activator, TCF7 functions as either an activator or a repressor depending on context and tissue specific expression of discrete isoforms [93]. For example, LEF1 expression is high in preosteoblasts and prevents their differentiation, while downregulation of LEF1 promotes differentiation of osteoblasts [94]. Similarly, TCF7 is highly expressed in hematopoietic progenitors, and activates expression of genes involved in self-renewal while inhibiting genes activated in differentiating cells [95]. Higher LEF1 and TCF7 expression in basally located cells (*i*.*e*. progenitors and precursors) and lower expression in elongated taste cells in the CVP epithelium suggests a similar role in the control of stemness and self-renewal to maintain taste cell homeostasis.

Wnt3 expression is virtually abolished in Krt5-β-catenin LOF mice compared with controls. Growth of intestinal organoids and their differentiation of Paneth cells depends on Paneth cell-derived Wnt3 [96], and absence of Wnt3 represses both expression of β-catenin and proliferation of gastric cancer cells [97]. Likewise, downregulation of Wnt3 in Krt5-β-catenin LOF mice is associated with a global run down of progenitors and loss of differentiated taste bud cells. Interestingly, intestinal stem cell homeostasis is not affected *in vivo* in the absence of Wnt3 [96] suggesting other Wnt signals are involved [96]. In tongue epithelium, Wnt10a [51] and Wnt10b [86] are expressed in basal progenitors of adult mice and in taste buds of new born mice, respectively. Wnt10a, like β-catenin, is required for proliferation of taste progenitors and differentiation of all three elongated taste cell types [51]. Interestingly, in our array data Wnt10a is slightly reduced in mutant CVP, although this difference is not significant (S1 Table). Further investigation of additional Wnt ligands, including Wnt3, are necessary to decipher the complexity of Wnt pathway function in taste cell homeostasis.

### Loss of taste buds in Krt5-β-catenin LOF mutant epithelium leads to reduced gustatory innervation

Interestingly, our findings suggest that reduced taste cells in mutants leads secondarily to a decline in gustatory innervation; specifically, taste buds become smaller and are lost in advance of reduced P2X2^+^ taste fibers. Loss of P2X2^+^ neurites may reflect pruning of fibers as taste bud cells are steadily diminished in the absence of β-catenin. Alternatively, gustatory fibers may persist, but instead downregulate expression of P2X2, which also would reduce afferent gustatory signals to the brain [66]. Taste buds are maintained by an intact innervation: gustatory nerve transection leads to taste bud degeneration, and if nerves re-innervate lingual epithelium, taste buds regenerate [52–54]. Interestingly, taste bud size is tightly correlated with innervation density in both mice and rats [98–100], suggesting taste buds may control their innervation in addition to a role for nerves in taste bud maintenance. Changes in P2X2^+^ neurites detected in mutant taste buds suggest gustatory fibers retract as their target taste cells are progressively lost. Under normal homeostasis, gustatory nerves may likewise undergo episodic remodeling as taste cells are lost, however these fibers do not retract but rather develop new contacts with newly generated taste cells. Thus, our data support a model where intact taste buds signal to nerves to maintain the gustatory innervation, as proposed previously [98]. This is a particularly intriguing finding, as the mechanisms by which taste bud cells rapidly turnover, yet maintain functional communication with sensory nerve fibers, are poorly understood.

### Anterior and posterior taste fields have differential sensitivity to loss of β-catenin

Deletion of β-catenin in Krt5^+^ progenitors results in a reduction in taste bud number and size in all taste fields examined, however, loss is more rapid and dramatic in FFP of the anterior tongue and SP compared with posterior CVP and FolP. While many, albeit smaller, taste buds remain in mutant CVP and FolP, virtually no FFP and SP taste buds are evident in mutants by 7 weeks. This pattern is similar to reports in the developing mouse tongue, where genetic deletion of Wnt10b or the LEF1 transcription factor leads to smaller FFP in embryos and loss of FFP in post-natal tongues, while the developing CVP is relatively unaffected [101]. Thus, different Wnt ligands and transcriptional regulators may operate in different taste fields. Consistent with this idea, LGR5 is expressed by CVP taste progenitors but is virtually absent in FFP [102, 103]. LGR5 is a Wnt target gene, as well as a receptor for R-Spondin ligands, which together augment and amplify canonical Wnt signaling [104, 105] suggesting that LGR5 signaling may blunt the impact of Krt5-β-catenin LOF in the posterior CVP. The temporal pattern of loss of *Shh*^+^ taste precursor cells also suggests differential molecular regulation of FFP versus CVP taste cell renewal. Despite the more rapid loss of FFP taste buds, *Shh*^+^ cells are readily evident in mutant taste buds, while in the CVP with a slower decline in buds, *Shh*^+^ cells are lost early on. One explanation is that Shh^-^ precursors may predominate in the CVP, *e*.*g*. MASH1^+^ cells [16, 106, 107] and Skn-1a^+^ cells [108]. Whether taste precursor subpopulations differ between taste fields and are differentially sensitive to loss of β-catenin remains to be tested.

### Differences in the rate of taste bud loss in anterior versus posterior taste fields results in taste modality-specific behavioral taste loss

In mutants, the rapid loss of FFP and SP taste buds versus the slower reduction in CVP and FolP correlates with early loss of sweet versus later reduced sensitivity to bitter perception, respectively. Although taste buds in all taste fields are sensitive to all taste qualities, data from gustatory nerve recordings and behavioral assays indicate that those in the FFP and SP innervated by the chorda tympani and greater superficial petrosal nerve, respectively, are more sensitive to sweet, whereas CVP taste buds innervated by the glossopharyngeal nerve, are better tuned to bitter stimuli [3–10, 109]. The contribution of FolP taste buds to the relative sensitivity to sweet and bitter is more difficult to evaluate as they are innervated by both chorda tympani and glossopharyngeal nerves [1]. However, multiple members of the bitter receptor family Tas2r are expressed predominantly in posterior CVP and FolP and more rarely in FFP, while the sweet and umami receptors, Tas1rs, are more highly expressed in FFP [2, 25]. Nonetheless, both bitter and sweet receptors are expressed in all taste fields, and thus we cannot rule out that early loss of sweet taste preference is due to concurrent downregulation of the sweet receptors in all taste fields. Similarly, Tas2Rs are expressed by all taste fields, and their expression may be less sensitive to Krt5-β-catenin LOF. These hypotheses remain to be tested. Interestingly, Wnt10a causes decreased taste bud maintenance, but does not lead to detectable alterations in taste sensitivity [51], suggesting Wnt10a may act partially redundantly with other canonical Wnt ligands such as Wnt3.

Sour perception is minimally affected in mutants, as aversion for citric acid is only slightly reduced compared to controls. Sour compounds are detected by Type III cells [29–31], making sour one of the basic taste modalities. However, acids are also detected by nociceptive free nerve endings within the general oral mucosa supplied by the trigeminal and glossopharyngeal nerves [110–112]. Thus, sour compounds activate trigeminal pathways in addition to gustatory pathways [112]. Our behavioral data support the interpretation that trigeminal detection may underlie the ability of mutant mice to detect acid when the gustatory system is impaired by β-catenin deletion. Similarly, trigeminal detection may also be responsible for the resilience of bitter perception that we observed at high concentrations [113].

In conclusion, our findings may be particularly relevant to drugs targeting the Wnt pathway in cancers. Numerous chemotherapeutics have been developed, several currently in clinical trials, to block elements of the Wnt pathway [114]. For instance, LGK-974 [115, 116] and ETC-159 (A*STAR, Phase I June 2015) are small molecular Porcupine (Porcn) inhibitors that block secretion of Wnt ligands, while OMP-54F28 and OMP-18-R5 are function-blocking antibodies for specific Wnt receptors–Frizzled proteins, that block Wnt ligand binding [117–125]. Our data in mice suggest a broad block of Wnt signaling, that would mimic Krt5-β-catenin LOF will likely affect taste buds and therefore taste function in patients. By contrast, more targeted inhibitors should only impact taste buds if, for example, a particular Frizzled receptor plays a role in taste bud cell renewal. To date, Fzd1 and 7, which both bind OMP-18-R5 [125], and Fzd3 are known to be expressed in adult mouse taste tissues [86], but their specific functions have yet to be investigated.

Many chemotherapy drugs cause patients to suffer taste dysfunction (dysgeusia), where the sense of taste persists but is unpleasantly distorted [126–128]. Patients experiencing dysgeusia have a significantly diminished quality of life, and ultimately poorer outcomes from cancer therapies [129–132]. Here we posit that an imbalance in the input of anterior versus posterior taste buds resulting from abrogation of Wnt signaling may underlie alterations in taste perception. Therefore, understanding the expression profile of Wnt pathway genes in taste tissues versus discrete cancers in patients treated with targeted drugs will help predict potential side effects on taste function, and ultimately aid in developing approaches to mitigate dysgeusia, including potentially topically reactivating the Wnt pathway [133].

## Materials and methods

### Ethics statement

All animal procedures were performed in an AAALAC-accredited facility in compliance with the Guide for the Care and Use of Laboratory Animals, Animal Welfare Act and Public Health Service Policy, and were approved by the Institutional Animal Care and Use Committee at the University of Colorado Anschutz Medical Campus.

### Animals and procedures

Mice were housed in sterile ventilated cages with *ad libitum* access to irradiated diet and hyperchlorinated reverse osmosis water delivered *via* an automatic watering system. The light cycle was from 6:00 to 20:00. All mouse lines were on a mixed background (FVB, 129Sv, C57Bl6). Both males and females between 8–12 weeks of age were used, and experimental and control animals selected based on genotype.

To delete β-catenin in Krt5^+^ progenitors, we created Krt5-β-catenin LOF mice, which carry Krt5rtTA (transcriptional activator rtTA controlled by the bovine Krt5 promoter [134]), tetOCre (tetO response element driving Cre recombinase [135]), and two alleles of Catnb^flox(exon2-6)^ [136]. To induce excision of exons 2–6 of β-catenin, Krt5-β-catenin LOF and control (lacking one or more engineered allele) mice, were fed the tetracycline analog doxycycline *ad libitum* in their chow (1 g/kg; Bio-Serv, Frenchtown, NJ) for 4 days, 2, 4 or 7 weeks, with free access to water.

To collect tongues for immunohistochemistry, mice were first anesthetized by i.p. injection of 250 mg/kg_body weight_ Avertin (2,2,2-Tribromoethanol). Ice-cold normal saline was perfused transcardially prior to perfusion with periodate-lysine-paraformaldehyde (PLP) fixative (75 mM l-Lysine monochloride, 1.6% formaldehyde, 10 mM sodium periodate) [137, 138]. Tongues were then immersed in PLP fixative for 3 h at 4°C on a rocker, and transferred to 20% sucrose (Fisher Scientific, Pittsburgh PA, USA) in 0.1 M phosphate buffer overnight at 4°C under constant gentle rocking. Samples were embedded in O.C.T. Compound (Tissue-Tek 4583, Sakura Finetek, Torrance CA, USA), frozen on dry ice and stored at -80°C.

To collect tongues for *in situ* hybridization, mice were euthanized by CO_2_ inhalation followed by cervical dislocation. The tongues were collected, rinsed in sterile ice-cold 1X Phosphate Buffered Saline (PBS: 29 mM NaH2PO4, 75 mM Na2HPO4, 154 mM NaCl) and embedded in O.C.T. Compound, frozen on dry ice and stored at -80°C.

To collect tongues for RT-PCR, mice were euthanized by CO_2_ inhalation followed by cervical dislocation. Tongues were collected and rinsed in ice-cold Normal Tyrode solution (140 mM NaCl, 5 mM KCl, 10 mM HEPES, 4 mM CaCl2, 10 mM glucose, 1 mM MgCl2, 1 mM sodium pyruvate, pH 7.4). CVP was harvested by cutting the epithelium with the underlying tissue (mesenchyme and Von Ebner’s glands) from the rest of the tongue (Fig 6B) or by peeling the epithelium after enzymatic digestion (Dispase II 3 mg/ml + Collagenase II 1 mg/ml in Normal Tyrode solution injected underneath the epithelium [15]) for 25 minutes at room temperature in calcium-free Tyrode and cutting the underlying tissue from the rest of the tongue (Fig 6C). Harvested tissues were dipped in RNAlater (Sigma-Aldrich) and immediately placed in a tube and frozen on dry ice. Samples were stored at -80°C.

### Immunohistochemistry

Immunostaining followed previously described procedures [15, 50]. Frozen 12 μm cryostat sections were collected on Superfrost Plus Slides (Fisher Scientific, Pittsburgh PA, USA). Primary and secondary antisera, amplification systems, and dilutions are listed in Table 1. Immunoreactivity for each antigen was absent when primary antibodies were omitted.

**Table 1:** Primary and secondary antibodies used for immunohistochemistry.

	Primary antibody	Source *Reference # /* *RRID #*	Dilution	Secondary Antibody	Source *Reference # /* *RRID #*	Dilution
Taste Cell Markers	Rabbit anti-NTPDase2	J. Sévigny, Université Laval, Canada (Bartel et al., 2006) *mN2-36*_*L*_ */* *N/A*	1/3000	Alexa Fluor 488 goat anti-rabbit IgG	ThermoFisher Scientific *A11008 /* *AB_143165*	1/1000
	Rabbit anti-PLCβ2	Santa Cruz *sc-206 /* *AB_632197*	1/1000			
	Rabbit anti-SNAP25	Sigma-Aldrich *S9684 /* *AB_261576*	1/6000			
Gustatory Innervation	Rabbit anti-P2X2	Alomone Labs APR-003 */* *AB_2040054*	1/500			
Cytokeratins	Rat anti-Krt8	Developmental Studies Hybridoma Bank *TROMA-I /* *AB_531826*	1/250	Alexa Fluor 546 or 488 goat anti-rat IgG	ThermoFisher Scientific *A11081 /* *AB_10563603* *A11006 /* *AB_2534074*	1/1000
Proliferation Marker	Rabbit anti-Ki67	Thermo Scientific *RM-9106-S /* *AB_2341197*	1/200	Anti-rabbit IgG biotinylated	Vector Labs *PK-6101 /* *AB_2336820*	1/500
Wnt signaling	Rabbit anti-TCF7	Thermo Scientific *MA5-14965 /* *AB_10983684*	1/100	Streptavidin Alexa Fluor 546 conjugate	ThermoFisher Scientific *S11225 /* *AB_2532130*	1/1000
	Mouse anti-β-catenin	Sigma-Aldrich *C7207 /* *AB_476865*	1/500	M.O.M. biotinylated anti-mouse IgG	Vector Labs *BMK-2202 /* *AB_2336833*	1/250
				Streptavidin Alexa Fluor 488 conjugate	ThermoFisher Scientific *S-11223 /* *AB_2336881*	1/600
	Rabbit anti-LEF1	Cell Signaling *2230 /* *AB_823558*	1/200	Alexa Fluor 488 goat anti-rabbit IgG	ThermoFisher Scientific *A11008 /* *AB_143165*	1/1000

#### β-catenin

Sections were thawed at room temperature, rehydrated in 0.1M PBS, and antigen retrieval was performed in 10 mM sodium citrate pH6 + 0.05% tween20 at 95°C for 15 min. Sections were incubated in blocking solution (2% normal goat serum, 1% bovine serum albumin, 0.3% Triton X100 in 0.1 M PBS, pH 7.3) for 45 min at room temperature. Blocking of endogenous avidin/biotin was performed with an Avidin/Biotin blocking kit (SP-2001, Vector Labs, Burlingame, CA, USA). Avidin solution was applied for 15 min, sections were washed and incubated in Biotin solution for 15 min and washed again. The M.O.M. kit from Vector Labs (BMK-2202) allows specific detection of the mouse anti-β-catenin antibody. M.O.M. Ig blocking reagent was applied for 1 h at room temperature, and sections were rinsed with 0.1 M PBS, incubated in M.O.M. diluent (protein concentrate diluted 1/14 in 0.1 M PBS) for 10 min at room temperature prior to incubation in primary antiserum diluted in M.O.M. diluent overnight at 4°C. Sections were washed, incubated in M.O.M. biotinylated anti-mouse IgG reagent prepared in M.O.M. diluent for 1 h at room temperature, rinsed again, and incubated with Streptavidin Alexa 488 diluted in PBS + 0.1% Triton X100 1 h at room temperature and protected from light. Nuclei were counterstained with DRAQ5 (abcam, Cambridge, MA, USA) diluted 1/8000 in 0.1 M Phosphate Buffer (PB: 29mM NaH2PO4, 75 mM Na2HPO4) for 30 min at room temperature, and washed in 0.1M PB. Sections were mounted under coverslips using Fluoromount G.

#### NTPdase2, PLCβ2, SNAP25, Krt8, P2X2, LEF1

Double immunohistochemistry was performed as previously described [15]. Here, NTPDase2, PLCβ2, SNAP25 or P2X2 rabbit antisera were used for double labeling with rat anti-Krt8 on PLP fixed sections, while LEF1 rabbit antiserum was used for double labeling with rat anti-Krt8 on fresh sections. Sections were thawed at room temperature, fixed 10 min in 4% PFA (LEF1 staining only), rehydrated in 0.1 M PBS, incubated for 1.5 h at room temperature in blocking solution (5% normal goat serum, 1% bovine serum albumin, 0.3% Triton X100 in 0.1 M phosphate buffered saline, pH 7.3), and incubated with primary antisera diluted in blocking solution overnight (except LEF1, 3 days) at 4°C. Sections were thoroughly washed in 0.1 M PBS + 0.1% Triton X100, and incubated with secondary antisera diluted in blocking solution for 1 h at room temperature and protected from light. Sections were counterstained with DRAQ5, and coverslipped with Fluoromount G.

#### Ki67, TCF7

Sections were thawed at room temperature, rehydrated in 0.1 M PBS, and antigen retrieval was performed as described above. After cooling to room temperature in antigen retrieval solution, sections were incubated in blocking solution (as above) for 1 h at room temperature, and incubated with Ki67 antiserum diluted in blocking solution overnight at 4°C. Sections were washed in 0.1 M PBS, and blocking of endogenous avidin/biotin was performed as above. Sections were incubated with anti-rabbit biotin-conjugated antibody diluted in 0.1 M PBS + 0.1% tween20 + 2.5% normal goat serum for 1 h at room temperature. Streptavidin-Alexa 546 diluted in 1% bovine serum albumin + 0.3% Triton X100 in 0.1 M PBS was then applied to the sections for 1 h at room temperature. Sections were counterstained with Sytox Green Nucleic Acid Stain (ThermoFisher, Carlsbad, CA, USA) diluted 1/30,000 in 0.1M PB pH 7.2 for 3 min at room temperature, washed, and coverslipped using Fluoromount G.

### *In situ* hybridization

Detection of mRNA encoding for *Shh* was performed as previously described [50]. Frozen 12 μm cryostat sections were collected on Superfrost Plus Slides (Fisher Scientific, Pittsburgh PA, USA). Antisense RNA probes were synthesized from a linearized plasmid containing a *Shh* cDNA insert [139], using digoxigenin-conjugated UTP. Sections were incubated in 4% PFA for 10 min at room temperature and rinsed in 0.1× PBS (14 mM NaCl, 0.3 mM KCl, 0.3 mM Na2HPO4, 0.2 mM KH2PO4). Anterior tongue sections required incubation in 2 μg/μl proteinase K for 2.5 min, were then rinsed in 0.1× PBS, incubated in 4% PFA for 10 min at room temperature and rinse in 0.1× PBS. CVP and anterior tongue sections were then incubated in triethanolamine solution (1.3% triethanolamine, 0.175% HCl 10 N, 0.25% acetic anhydride), rinsed in 0.1× PBS, incubated in hybridization solution (50% formamide, 5× SCC (750 mM NaCl, 75 mM sodium citrate dihydrate), 5× Denhardt’s solution (0.1% Ficoll, 0.1% polyvinylpyrrolidone, and 0.1% bovine serum albumin, 500 μg/ml salmon sperm DNA and 250 μg/ml tRNA) for 2 h at room temperature, then with the RNA probe in hybridization solution overnight at 65°C in a moist chamber. Sections were incubated 90 min at 65°C in 0.2× SSC (30 mM NaCl, 3 mM sodium citrate dihydrate), then in Buffer 2T (0.1 M Tris, 0.15 M NaCl, 1% Roche Applied Science blocking reagent 11096176001, 0.05% Tween 20) for 1 h at room temperature, and incubated with Alkaline Phosphatase-coupled anti-digoxigenin antibody diluted 1/600 in Buffer 2T overnight in a moist chamber at 4°C. Sections were treated with NBT/BCIP solution (Roche Applied Science, 11681451001) in 0.1 M Tris-HCl pH 9.5 + 0.1 M NaCl + 50 mM MgCl_2_ at room temperature until desired staining is obtained. Reaction was blocked in 10 mM Tris-HCl pH 8 + 1 mM EDTA for at least 10 min, and slides were coverslipped with Fluoromount G.

### Image acquisition and analysis

Confocal fluorescence images were acquired using a Leica TCS SP5 II laser-scanning confocal microscope and LASAF software. Immunolabeled cells were tallied by analyzing both 0.76 μm optical sections and compressed z-stacks of 14 optical sections. Nomarski images were acquired using a Zeiss Axioplan 2 microscope, camera and software. All sections of the CVP, except the first and last sections which were excluded as they generally contain incomplete trenches, were analyzed. For the anterior tongue, 12 μm serial sections were cut into 6 sets such that sections on each slide were separated by 72 μm. FFP were analyzed in the 2^nd^ through the 10^th^ section, while the 1^st^ section was omitted due to the curved nature of the tongue surface and difficulty in interpreting non-transverse sections through FFP. Thus, we analyzed FFP in a region representing 648 μm of the anterior tongue starting ~72 μm from the tongue tip.

Proliferative index in the CVP was calculated as previously published [15, 59]. The number of Ki67^+^ basal cells was divided by the number of Sytox Green^+^ basal cells along the basement membrane, within the portion of the CVP trenches housing taste buds.

CVP size was determined by measuring the total cross sectional area between the two trenches, including the epithelium and mesenchyme in a single tissue section, using ImageJ (NIH). The depth of both CVP trenches per section was measured from the bottom of each trench to a line connecting the lingual surface at either side of the trench (See Fig 1D).

The total area per taste bud occupied by NTPdase2-immunofluorescence signal was used as a proxy to estimate relative Type I cell populations across treatments and was quantified using ImageJ (NIH). The measurement unit was calibrated from pixel to μm using the *Set Scale* function. Grayscale z-stacks images of NTPDase2 fluorescence (green channel) were converted to binary images using the *Threshold* function. Each Krt8^+^ (red channel) taste bud profile was outlined with the *Freehand selections* tool to define taste bud area (*Area* in *Set Measurements*, in μm^2^). The density of NTPDase2^+^ signal within the Krt8^+^ taste bud area was measured with the *Area fraction* function (in *Set Measurements*) to define the percent of the taste bud area occupied by NTPDase2^+^ pixels. The total area per taste bud occupied by NTPDase2^+^ signal (μm^2^) per taste bud was determined by multiplying the *Area* by the *Area fraction*.

P2X2^+^ gustatory innervation within Krt8^+^ taste buds was measured using our in-house MATLAB (Mathworks, Natick, MA) toolbox, *imstack*, adapted from [100]. Briefly, the user defined the Krt8^+^ taste bud region of interest (ROI) throughout the entire 14 optical section z stack. A threshold value for the pixels in this ROI was automatically generated using Otsu’s method [140]. This threshold was then adjusted to accommodate signal-to-noise ratios across the entire experimental image dataset and eliminate noise. Finally, pixels with intensity values above the adjusted threshold value were designated as positive for P2X2^+^ signal. The total number P2X2^+^ positive pixels were then counted within a given ROI to determine number of P2X2^+^ pixels per taste bud profile.

### Real-time RT-PCR

Total RNA was extracted using the RNeasy Plus Micro kit (Qiagen, 74034). Nanodrop (ThermoFisher Scientific) was used to measure the amount of RNA extracted and High Sensitivity RNA ScreenTape Station (Agilent technologies) was used to assess RNA integrity.

#### Wnt signaling pathway gene expression array

cDNA was synthesized from 100 ng total RNA subjected to preliminary genomic DNA elimination followed by reverse transcription and preamplification of cDNA targets by using the RT^2^ PreAMP cDNA Synthesis Kit (Qiagen, 330451) and RT^2^ PreAMP Pathway Primer Mix (Qiagen, 330241) according to manufacturer’s instructions. Real-Time PCR was run by using the WNT Signaling Pathway RT^2^ Profiler PCR Array (Qiagen, PAMM-043ZC) and RT^2^ SYBR Green ROX qPCR Mastermix (Qiagen, 330522) according to manufacturer’s instructions with an Applied Biosystems StepOnePlus real-time thermocycler. Data analysis was performed following the Qiagen RT^2^ Profiler PCR Array Data Analysis v3.5 Handbook, and by using the online Qiagen Data Analysis Center. All samples passed data quality control integrated to the array plates (PCR Array Reproducibility, RT Efficiency, absence of Genomic DNA Contamination). Specificity of each assay was confirmed by the presence of a single peak in the melt curve. C_T_ values were determined by setting an automated baseline and the threshold manually at 0.08 across all array runs. Lower limit of detection (“C_T_ cut-off”) was set at 35. Automatic Selection from Full Plate was used as the normalization method resulting in the selection of Nfatc1, Fzd3, Frat1, Kremen1 and Ctbp1 for normalization genes.

#### Expression of individual genes

cDNA was synthesized from 100 ng total RNA by using iScript cDNA Synthesis Kit (Biorad) according to manufacturer’s protocol. cDNA equivalent of 2.5 ng total RNA, 250–300 nM of the forward and reverse primers were mixed with Power SYBR Green PCR Master Mix (Applied Biosystems). Real-Time PCR was run in a StepOnePlus Real-Time thermocycler (Applied Biosystems) and consisted of forty 95°C/15 s-60°C/60 s cycles. mRNA levels were normalized to RPL19 (Fig 6B) or β-actin (Fig 6C) mRNA levels. Primers sequences are shown in Table 2.

**Table 2:** Primers sequences used in real-time RT-PCR.

Gene name	Refseq	Forward primer (5’-3’)	Reverse primer (5’-3’)
RPL19	NM_009078.2	GGTCTGGTTGGATCCCAATG	CCCGGGAATGGACAGTCA
β-actin	NM_007393	ACCAACTGGGACGATATGGAGAAGA	TACGACCAGAGGCATACAGGGACAA
Wnt3	NM_009521	CTTCTAATGGAGCCCCACCT	GAGGCCAGAGATGTGTACTGC
LEF1	NM_010703	TATGAACAGCGACCCGTACA	CTCGTCGCTGTAGGTGATGA
TCF7	NM_009331	CAATCTGCTCATGCCCTACC	TAGAGTGGAGAAAGCTGGGG

http://mouseprimerdepot.nci.nih.gov

The comparative ΔΔC_T_ method was used for relative quantification of gene expression [141]. RPL19 was used as a housekeeping gene to compare the relative expression of genes of interests between control and mutant mice at 2 weeks (Fig 6B). Because RPL19 average raw C_T_ value was 1.6 cycles higher in the mesenchyme than in the epithelium, β-actin was used as the housekeeping gene to compare relative expression of genes of interest between CVP epithelium and mesenchyme (Fig 6C).

### Two-bottle preference test

Preference for saccharine was tested in a 48 h two-bottle test paradigm, as previously described [66, 73, 142]. Mice were individually housed in regular ventilated cages with ad libitum access to chow. Lickometer non-graduated glass bottles with stainless steel sipper tubes were used. First, mice were trained to drink out of bottles instead of the regular “lixit” water supply. Mice were given two bottles of deionized water for 48 h; the left-right position of the bottles was switched after 24 h. Mice were then given one bottle of water and one bottle of saccharine (3 mM in deionized water) for 48 h; Volume of liquid consumed was measured after 24 h, the bottles refilled if necessary, and left-right position switched to control for side preference. The amount of solution in each bottle was measured by weight and converted to volume (1 g = 1 ml) after the first and second 24 h, and summed to obtain the total volume consumed in 48 h. The preference ratio was calculated by dividing the total volume of saccharine by the total volume of water and saccharine consumed.

### Brief-access lickometer test

Four Davis Rig MS-160 lickometers (DiLog Instruments, Inc., Tallahassee, FL, USA) were used to test taste perception of control and mutant mice. Detailed protocol and operating procedures have been previously described [73, 143, 144]. Mice were tested individually and randomly allocated to one of the four lickometers each day. Black paper sheets were placed on each side of the lickometer chamber and the chambers area was reduced by half to reduce distraction from surroundings and exploration, respectively. Training and test sessions were limited to 30 min. Brief-access consisted of a shutter opening to give mice access to a bottle of tastant during a 5 s trial (counted from first lick), then the shutter closed for 7.5 s, meanwhile the next bottle was moved into place for the next trial. Each bottle was presented 5 times. Mice were water deprived for 18 h prior to training or testing to motivate them to drink during the short allocated time. Mice were trained for three consecutive days to drink water in the lickometer setup. On the first day, the shutter was continuously open to make one bottle of water freely available for 30 min. On the second and third days, 4 bottles of water were placed on the mobile rack, and presented for 5 s each, repeated 5 times. Tastants were tested on the following days, one range of concentration of a single tastant per day. Three taste qualities were tested: Sweet—SC45647 (3, 10 and 100 μM), Bitter–denatonium (0.01, 0.1 and 0.5 mM) and Sour–citric acid (1, 30, 100 mM). All solutions were made in deionized water obtained from in-house commercial filtering system. One test session consisted of five trial blocks with one bottle of deionized water and three concentrations of the tastant to be tested, presented in random order [73]. To maintain motivation during testing of bitter and sour stimuli, which are aversive, one bottle of 10 μM SC45647 was included in the presentation protocol. First and last blocks were not included in the data analysis as thirst-induced high motivational state during the first block may induce maximal licking for all solutions, whereas the last block may reflect post-ingestive detection of tastants. Results are expressed as the lick ratio (average number of licks triggered by a tastant stimulus divided by the average number of licks triggered by water).

### Statistical analysis

Statistical analyses were performed using SigmaStat (Systat Software, San Jose, CA, USA) or Graphpad Prism. Normal distribution and equal variances between groups were assessed to determine whether to run a parametric or non-parametric test. A Mann-Whitney test or Student’s t-test was run to determine statistical differences established with a minimum confidence interval of 95%. Non-parametric data are represented as scatter plots (individual symbols) with medians, 1^st^ and 3^rd^ quartiles, while parametric data are represented as scatter plots (individual symbols) with means ± SEM. Two-sample chi-square was applied to assess differences in data distribution of control vs mutant groups; Chi^2^ and p values are shown with the respective graphs.

## Supporting information

S1 Fig β-catenin immunostaining is reduced in basal keratinocytes and taste buds in Krt5-β-catenin LOF mice. Circumvallate papillae (CVP) and fungiform papillae (FFP) were harvested from *Krt5rtTA;tetOCre;Catnb*^*flox(exon2-6)*^ mice fed doxycycline chow for 4 weeks, and cryosections immunostained for β-catenin. Perigemmal basal cells (white arrowheads), including progenitor cells, and taste bud cells (*) in control CVP and FFP express β-catenin (**A**, **B**), while β-catenin immunostaining was lost in CVP and FFP perigemmal basal cells (**A’**,**B’**, yellow arrowheads) and in non-taste cells (**A’**,**B’**, white arrows) in mutant mice. In both CVP and FFP a smaller number of taste bud cells (*) are still β-catenin^+^ and are likely cells older than 4 weeks. N = 3 control and 3 mutant mice. Representative images are compressed z-stacks. Nuclei were counterstained with DRAQ5 (magenta). Dotted lines delineate the basement membrane. Taste buds are marked with asterisks. Scale bars = 20 μm.
(TIF)

S2 Fig Krt5-β-catenin LOF reduces the number and size of taste buds in foliate papillae (FolP) and soft palate (SP). The number and size of taste buds was significantly reduced in the FolP (**A-C**) and SP (**D-F**) of mutant mice compared to those of controls. Data are represented as scatter plots (individual symbols), and median with 1^st^ and 3^rd^ quartile (blue bars. Mann & Whitney test). **(B)** 33 vs 29 FolP trench profiles from 3 control mice vs 3 mutant mice, respectively; **(C)** 89 vs 38 FolP taste bud profiles from 3 control mice vs 3 mutant mice, respectively; **(E)** 57 vs 52 SP sections from 3 control mice vs 3 mutant mice, respectively; **(F)** 25 vs 9 palate taste bud profiles from 3 control mice vs 3 mutant mice, respectively. TB: taste bud. Representative images are compressed z-stacks. Nuclei were counterstained with DRAQ5 (magenta). Dotted lines delineate the basement membrane. Scale bars = 20 μm.
(TIF)

S3 Fig β-catenin deletion in Krt5^+^ progenitors reduces all 3 cells types in FFP. **(A)** NTPDase2 (green) marks the membranes of Type I glial-like taste cells, as well as a subset of mesenchymal cells adjacent to the CVP epithelium (white arrowheads). In the anterior tongue, the area of NTPdase2-immunostaining (green) per FFP Krt8^+^ taste bud profile was reduced in mutants (black bars) compared to controls (white bars) at all time points. **(B)** FFP taste buds of mutant mice fed doxycycline for 2, 4 and 7 weeks had fewer PLCβ2^+^ Type II cells (green) compared to controls. **(C)** The number of SNAP25^+^ Type III cells (green) per taste bud profile did not differ from controls at 2 weeks, but was significantly reduced at 4 and 7 weeks of β-catenin deletion. Data are represented as taste bud size distribution (Two-sample chi-square for trend). Sample sizes: **(A)** 4–51 FFP taste bud profiles; **(B)** 6–117 FFP taste bud profiles; **(C)** 4–51 FFP taste bud profiles, from 3–4 controls and 3–4 mutants per time point. Note: low numbers of taste buds observed represent those measured at 7 weeks, when few FFP taste buds remained in all animals. TB: taste bud. Representative images are compressed z-stacks. Nuclei were counterstained with DRAQ5 (magenta). Dotted lines delineate the basement membrane. Scale bars = 20 μm.
(TIF)

S4 Fig Krt5-β-catenin LOF mice show reduced preference for saccharine and have significant weight loss compared to controls. **(A)** The weight of control and mutant mice was measured at 2 weeks and 7 weeks of doxycycline chow. Beta-catenin deletion in Krt5^+^ progenitor cells was associated with significant weight loss. Data are represented as scatter plots (individual symbols), and mean ± SEM (Student’s t-test). N = 10 control mice and 6 mutant mice. **(B)** Control and mutant mice were subjected to a preference test between water and an appetitive concentration of a sweet compound (3 mM saccharine). Control mice showed a strong preference for 3 mM saccharine over water in a 48 h two-bottle preference test, while after 4 weeks of doxycycline chow Krt5-β-catenin LOF mice did not discriminate saccharine from water. The preference score is the ratio of volume of saccharine consumed divided by the total volume of saccharine plus water consumed; green dash line marks a 50% preference score, *i*.*e*. absence of preference for saccharine over water. Data are represented as scatter plots (individual symbols), and mean ± SEM (Student’s t-test). N = 7 control mice and 10 mutant mice.
(TIF)

S1 Table Wnt signaling pathway real-time PCR array data. (DOCX)
