# Supplementary figures and images for: β-catenin is required for taste bud cell renewal and behavioral taste perception in adult mice
Source: PLoS Genet. 2017 Aug 28;13(8):e1006990. doi: 10.1371/journal.pgen.1006990 (PMC5591015; doi:10.1371/journal.pgen.1006990)

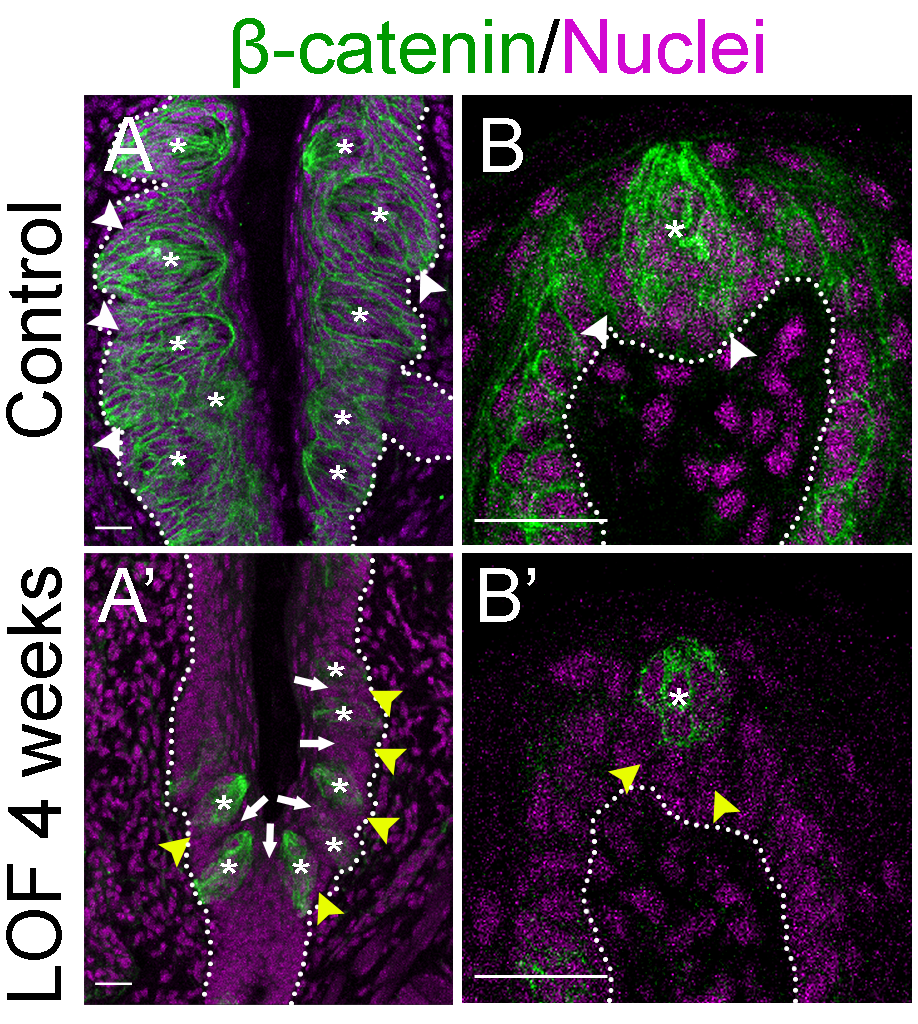

Supplement: S1 Fig — Circumvallate papillae (CVP) and fungiform papillae (FFP) were harvested from Krt5rtTA;tetOCre;Catnbflox(exon2-6) mice fed doxycycline chow for 4 weeks, and cryosections immunostained for β-catenin. Perigemmal basal cells (white arrowheads), including progenitor cells, and taste bud cells (*) in control CVP and FFP express β-catenin (A, B), while β-catenin immunostaining was lost in CVP and FFP perigemmal basal cells (A’,B’, yellow arrowheads) and in non-taste cells (A’,B’, white arrows) in mutant mice. In both CVP and FFP a smaller number of taste bud cells (*) are still β-catenin+ and are likely cells older than 4 weeks. N = 3 control and 3 mutant mice. Representative images are compressed z-stacks. Nuclei were counterstained with DRAQ5 (magenta). Dotted lines delineate the basement membrane. Taste buds are marked with asterisks. Scale bars = 20 μm. (TIF) [file pgen.1006990.s001.tif]

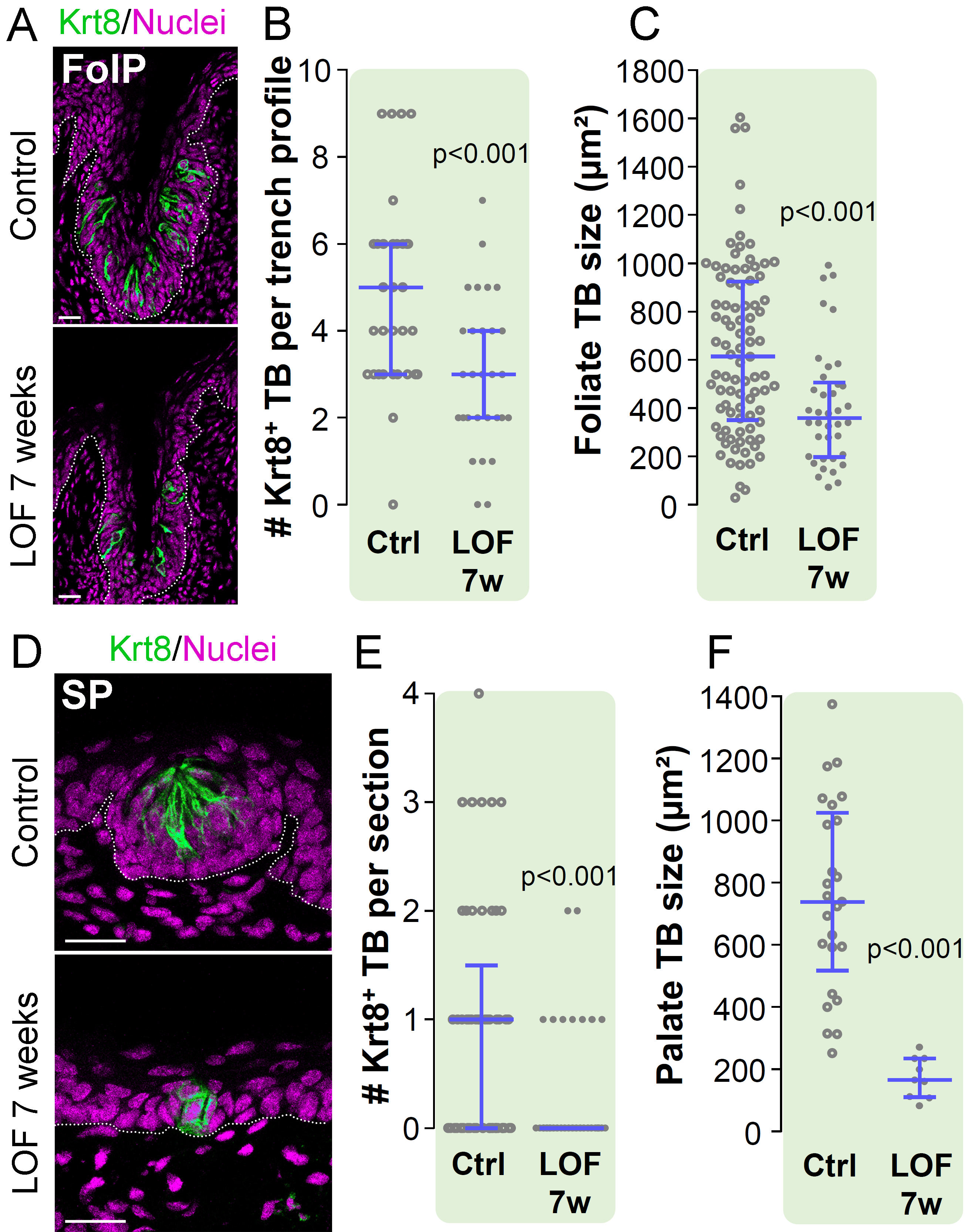

Supplement: S2 Fig — The number and size of taste buds was significantly reduced in the FolP (A-C) and SP (D-F) of mutant mice compared to those of controls. Data are represented as scatter plots (individual symbols), and median with 1st and 3rd quartile (blue bars. Mann & Whitney test). (B) 33 vs 29 FolP trench profiles from 3 control mice vs 3 mutant mice, respectively; (C) 89 vs 38 FolP taste bud profiles from 3 control mice vs 3 mutant mice, respectively; (E) 57 vs 52 SP sections from 3 control mice vs 3 mutant mice, respectively; (F) 25 vs 9 palate taste bud profiles from 3 control mice vs 3 mutant mice, respectively. TB: taste bud. Representative images are compressed z-stacks. Nuclei were counterstained with DRAQ5 (magenta). Dotted lines delineate the basement membrane. Scale bars = 20 μm. (TIF) [file pgen.1006990.s002.tif]

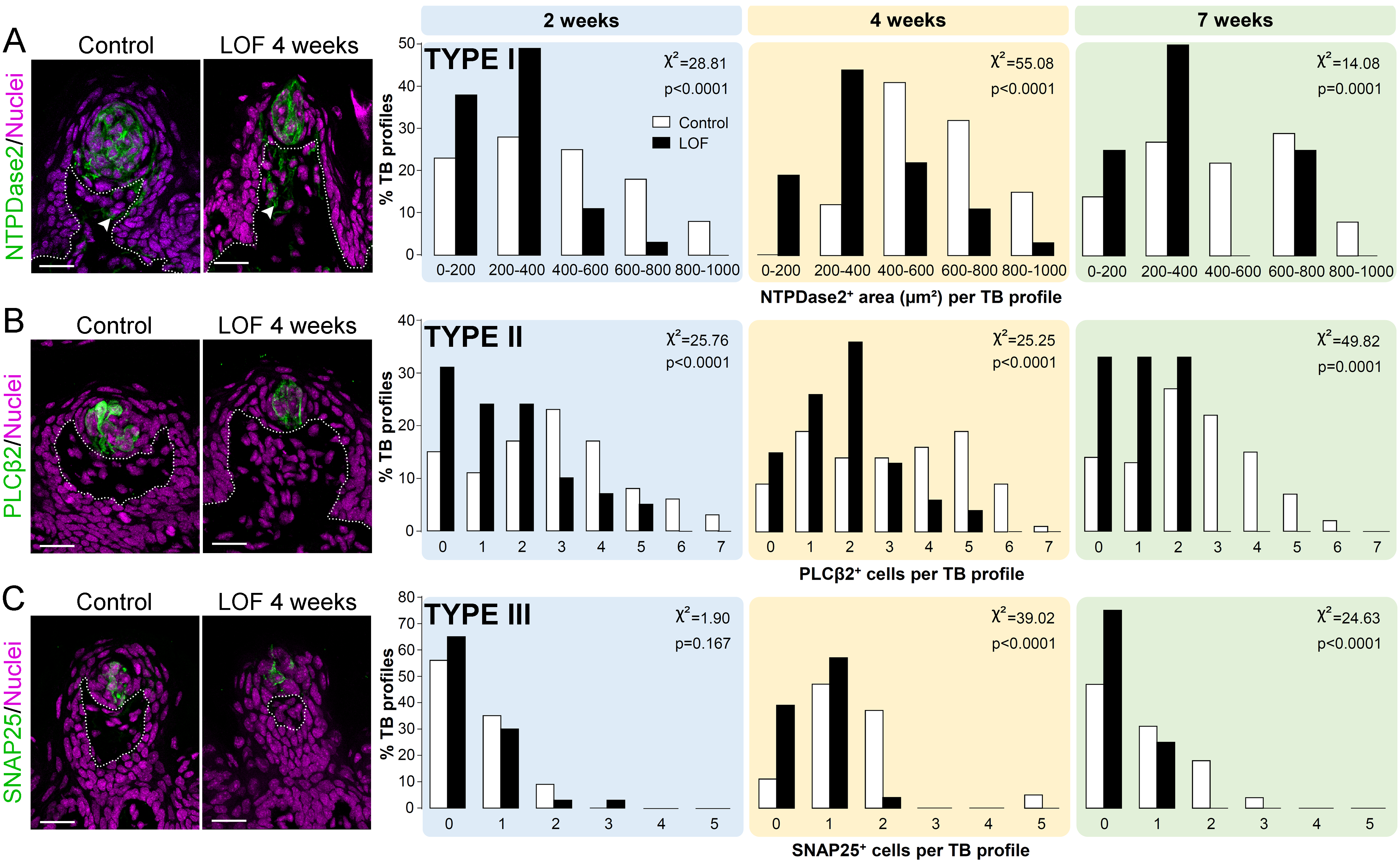

Supplement: S3 Fig — (A) NTPDase2 (green) marks the membranes of Type I glial-like taste cells, as well as a subset of mesenchymal cells adjacent to the CVP epithelium (white arrowheads). In the anterior tongue, the area of NTPdase2-immunostaining (green) per FFP Krt8+ taste bud profile was reduced in mutants (black bars) compared to controls (white bars) at all time points. (B) FFP taste buds of mutant mice fed doxycycline for 2, 4 and 7 weeks had fewer PLCβ2+ Type II cells (green) compared to controls. (C) The number of SNAP25+ Type III cells (green) per taste bud profile did not differ from controls at 2 weeks, but was significantly reduced at 4 and 7 weeks of β-catenin deletion. Data are represented as taste bud size distribution (Two-sample chi-square for trend). Sample sizes: (A) 4–51 FFP taste bud profiles; (B) 6–117 FFP taste bud profiles; (C) 4–51 FFP taste bud profiles, from 3–4 controls and 3–4 mutants per time point. Note: low numbers of taste buds observed represent those measured at 7 weeks, when few FFP taste buds remained in all animals. TB: taste bud. Representative images are compressed z-stacks. Nuclei were counterstained with DRAQ5 (magenta). Dotted lines delineate the basement membrane. Scale bars = 20 μm. (TIF) [file pgen.1006990.s003.tif]

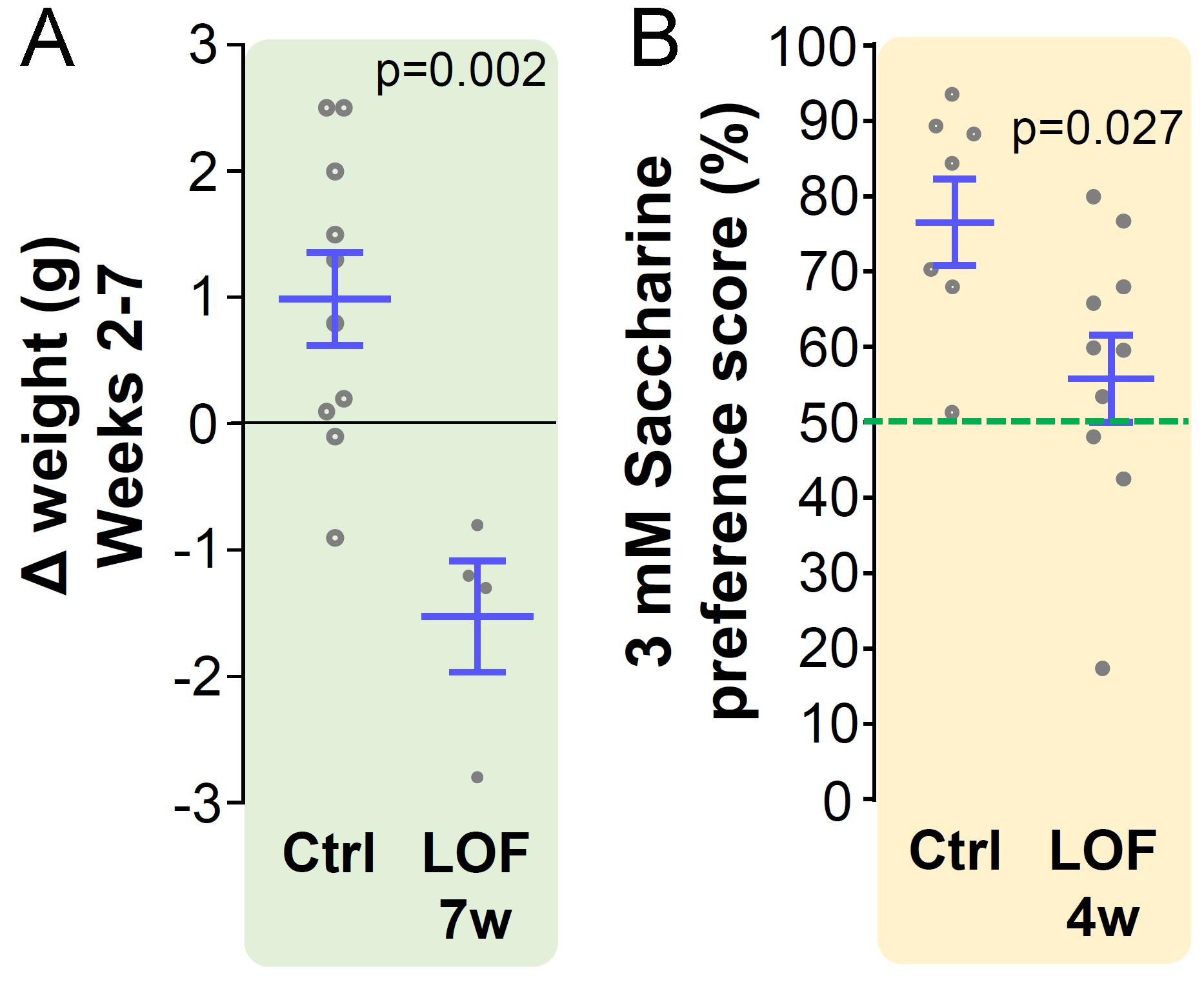

Supplement: S4 Fig — (A) The weight of control and mutant mice was measured at 2 weeks and 7 weeks of doxycycline chow. Beta-catenin deletion in Krt5+ progenitor cells was associated with significant weight loss. Data are represented as scatter plots (individual symbols), and mean ± SEM (Student’s t-test). N = 10 control mice and 6 mutant mice. (B) Control and mutant mice were subjected to a preference test between water and an appetitive concentration of a sweet compound (3 mM saccharine). Control mice showed a strong preference for 3 mM saccharine over water in a 48 h two-bottle preference test, while after 4 weeks of doxycycline chow Krt5-β-catenin LOF mice did not discriminate saccharine from water. The preference score is the ratio of volume of saccharine consumed divided by the total volume of saccharine plus water consumed; green dash line marks a 50% preference score, i.e. absence of preference for saccharine over water. Data are represented as scatter plots (individual symbols), and mean ± SEM (Student’s t-test). N = 7 control mice and 10 mutant mice. (TIF) [file pgen.1006990.s004.tif]
