# Supplementary material for: β-catenin is required for taste bud cell renewal and behavioral taste perception in adult mice
Source: PLoS Genet. 2017 Aug 28;13(8):e1006990. doi: 10.1371/journal.pgen.1006990 (PMC5591015; doi:10.1371/journal.pgen.1006990)
Supplement: S1 Table — (DOCX) [file pgen.1006990.s005.docx]

**S1 Table. Wnt Signaling Pathway Real-Time PCR Array data**

***Sorted by fold regulation***

| **Gene name** | **Description** | **Unigene** | **Refseq** | **Fold**  **Regulation** | **p-value** |
| --- | --- | --- | --- | --- | --- |
| Wnt3 | Wingless-related MMTV integration site 3 | Mm.159091 | NM_009521 | -109.1163 | 0.000173 |
| Ctnnb1 | Catenin (cadherin associated protein), beta 1 | Mm.291928 | NM_007614 | -5.8606 | 0.039424 |
| Fzd1 | Frizzled homolog 1 (Drosophila) | Mm.246003 | NM_021457 | -3.192 | 0.43124 |
| Wnt7b | Wingless-related MMTV integration site 7B | Mm.306946 | NM_009528 | -2.7135 | 0.798107 |
| Wnt9a | Wingless-type MMTV integration site 9A | Mm.218794 | NM_139298 | -2.7019 | 0.843866 |
| Fzd8 | Frizzled homolog 8 (Drosophila) | Mm.184289 | NM_008058 | -2.6042 | 0.934413 |
| Lef1 | Lymphoid enhancer binding factor 1 | Mm.255219 | NM_010703 | -2.3653 | 0.039284 |
| Rhou | Ras homolog gene family, member U | Mm.168257 | NM_133955 | -2.2906 | 0.920277 |
| Fzd6 | Frizzled homolog 6 (Drosophila) | Mm.4769 | NM_008056 | -2.2862 | 0.472544 |
| Wif1 | Wnt inhibitory factor 1 | Mm.32831 | NM_011915 | -2.1939 | 0.067763 |
| Vangl2 | Vang-like 2 (van gogh, Drosophila) | Mm.36148 | NM_033509 | -2.1611 | 0.22507 |
| Ep300 | E1A binding protein p300 | Mm.258397 | NM_177821 | -2.1073 | 0.85848 |
| Tcf7 | Transcription factor 7, T-cell specific | Mm.31630 | NM_009331 | -2.0596 | 0.000314 |
| Wnt10a | Wingless related MMTV integration site 10a | Mm.5130 | NM_009518 | -1.9184 | 0.537276 |
| Mmp7 | Matrix metallopeptidase 7 | Mm.4825 | NM_010810 | -1.7537 | 0.092557 |
| Nkd1 | Naked cuticle 1 homolog (Drosophila) | Mm.30219 | NM_027280 | -1.7352 | 0.014712 |
| Rhoa | Ras homolog gene family, member A | Mm.757 | NM_016802 | -1.7267 | 0.70549 |
| Dkk1 | Dickkopf homolog 1 (Xenopus laevis) | Mm.214717 | NM_010051 | -1.7203 | 0.970153 |
| Fzd4 | Frizzled homolog 4 (Drosophila) | Mm.86755 | NM_008055 | -1.7156 | 0.722114 |
| Daam1 | Dishevelled associated activator of morphogenesis 1 | Mm.474935 | NM_172464 | -1.7084 | 0.529445 |
| Btrc | Beta-transducin repeat containing protein | Mm.119717 | NM_009771 | -1.6029 | 0.912582 |
| Wnt5a | Wingless-related MMTV integration site 5A | Mm.287544 | NM_009524 | -1.6002 | 0.056766 |
| Ctnnbip1 | Catenin beta interacting protein 1 | Mm.299735 | NM_023465 | -1.5501 | 0.318734 |
| Tcf7l1 | Transcription factor 7-like 1 (T-cell specific, HMG box) | Mm.440067 | NM_009332 | -1.5414 | 0.656847 |
| Lrp6 | Low density lipoprotein receptor-related protein 6 | Mm.321990 | NM_008514 | -1.5205 | 0.604974 |
| Sfrp1 | Secreted frizzled-related protein 1 | Mm.281691 | NM_013834 | -1.5016 | 0.66985 |
| Jun | Jun oncogene | Mm.275071 | NM_010591 | -1.4966 | 0.890874 |
| Fosl1 | Fos-like antigen 1 | Mm.6215 | NM_010235 | -1.4914 | 0.33896 |
| Fzd9 | Frizzled homolog 9 (Drosophila) | Mm.6256 | NM_010246 | -1.4482 | 0.650101 |
| Myc | Myelocytomatosis oncogene | Mm.2444 | NM_010849 | -1.4446 | 0.593277 |
| Foxn1 | Forkhead box N1 | Mm.4496 | NM_008238 | -1.4166 | 0.037657 |
| Axin2 | Axin2 | Mm.71710 | NM_015732 | -1.4147 | 0.214857 |
| Dkk3 | Dickkopf homolog 3 (Xenopus laevis) | Mm.55143 | NM_015814 | -1.3741 | 0.751949 |
| Apc | Adenomatosis polyposis coli | Mm.384171 | NM_007462 | -1.3731 | 0.189294 |
| Ccnd2 | Cyclin D2 | Mm.333406 | NM_009829 | -1.3516 | 0.324436 |
| Nlk | Nemo like kinase | Mm.9001 | NM_008702 | -1.3141 | 0.005784 |
| Sox17 | SRY-box containing gene 17 | Mm.279103 | NM_011441 | -1.3057 | 0.519571 |
| Wnt5b | Wingless-related MMTV integration site 5B | Mm.321818 | NM_009525 | -1.2992 | 0.354011 |
| Fzd5 | Frizzled homolog 5 (Drosophila) | Mm.150813 | NM_022721 | -1.2618 | 0.545532 |
| Csnk1a1 | Casein kinase 1, alpha 1 | Mm.26908 | NM_146087 | -1.2452 | 0.059202 |
| Ppard | Peroxisome proliferator activator receptor delta | Mm.328914 | NM_011145 | -1.2393 | 0.101587 |
| Csnk2a1 | Casein kinase 2, alpha 1 polypeptide | Mm.23692 | NM_007788 | -1.232 | 0.769848 |
| Axin1 | Axin 1 | Mm.23684 | NM_009733 | -1.2142 | 0.25539 |
| Bcl9 | B-cell CLL/lymphoma 9 | Mm.226175 | NM_029933 | -1.2022 | 0.009513 |
| Lrp5 | Low density lipoprotein receptor-related protein 5 | Mm.274581 | NM_008513 | -1.1865 | 0.404926 |
| Wnt11 | Wingless-related MMTV integration site 11 | Mm.22182 | NM_009519 | -1.1862 | 0.382355 |
| Dvl2 | Dishevelled 2, dsh homolog (Drosophila) | Mm.5114 | NM_007888 | -1.1803 | 0.730529 |
| Wnt3a | Wingless-related MMTV integration site 3A | Mm.1367 | NM_009522 | -1.174 | 0.522624 |
| Wnt8b | Wingless related MMTV integration site 8b | Mm.88365 | NM_011720 | -1.1596 | 0.701231 |
| Pitx2 | Paired-like homeodomain transcription factor 2 | Mm.246804 | NM_011098 | -1.1387 | 0.881615 |
| Dvl1 | Dishevelled, dsh homolog 1 (Drosophila) | Mm.3400 | NM_010091 | -1.1331 | 0.484771 |
| Ruvbl1 | RuvB-like protein 1 | Mm.42195 | NM_019685 | -1.1218 | 0.301791 |
| Dixdc1 | DIX domain containing 1 | Mm.82598 | NM_178118 | -1.1164 | 0.907455 |
| Wnt4 | Wingless-related MMTV integration site 4 | Mm.20355 | NM_009523 | -1.0508 | 0.978068 |
| Frat1 | Frequently rearranged in advanced T-cell lymphomas | Mm.4573 | NM_008043 | -1.0382 | 0.274603 |
| Ctbp1 | C-terminal binding protein 1 | Mm.240076 | NM_013502 | -1.0248 | 0.764843 |
| Prickle1 | Prickle homolog 1 (Drosophila) | Mm.150314 | NM_001033217 | -1.01 | 0.619611 |
| Fzd3 | Frizzled homolog 3 (Drosophila) | Mm.214687 | NM_021458 | 1.0007 | 0.968589 |
| Gsk3b | Glycogen synthase kinase 3 beta | Mm.394930 | NM_019827 | 1.008 | 0.920974 |
| Fzd2 | Frizzled homolog 2 (Drosophila) | Mm.36416 | NM_020510 | 1.0131 | 0.94147 |
| Kremen1 | Kringle containing transmembrane protein 1 | Mm.209989 | NM_032396 | 1.015 | 0.736121 |
| Nfatc1 | Nuclear factor of activated T-cells, cytoplasmic, calcineurin-dependent 1 | Mm.329560 | NM_016791 | 1.0463 | 0.325824 |
| Fbxw11 | F-box and WD-40 domain protein 11 | Mm.28017 | NM_134015 | 1.0532 | 0.581796 |
| Aes | Amino-terminal enhancer of split | Mm.180013 | NM_010347 | 1.0988 | 0.496949 |
| Tle1 | Transducin-like enhancer of split 1, homolog of Drosophila E(spl) | Mm.278444 | NM_011599 | 1.1313 | 0.158722 |
| Pygo1 | Pygopus 1 | Mm.273605 | NM_028116 | 1.1384 | 0.480802 |
| Wnt2b | Wingless related MMTV integration site 2b | Mm.10740 | NM_009520 | 1.1742 | 0.492405 |
| Mapk8 | Mitogen-activated protein kinase 8 | Mm.21495 | NM_016700 | 1.1753 | 0.168594 |
| Fbxw4 | F-box and WD-40 domain protein 4 | Mm.254739 | NM_013907 | 1.1808 | 0.138696 |
| Fzd7 | Frizzled homolog 7 (Drosophila) | Mm.297906 | NM_008057 | 1.2232 | 0.155797 |
| Ccnd1 | Cyclin D1 | Mm.273049 | NM_007631 | 1.2258 | 0.285213 |
| Porcn | Porcupine homolog (Drosophila) | Mm.443425 | NM_023638 | 1.291 | 0.315221 |
| Wnt6 | Wingless-related MMTV integration site 6 | Mm.268282 | NM_009526 | 1.3246 | 0.405535 |
| Dab2 | Disabled homolog 2 (Drosophila) | Mm.240830 | NM_023118 | 1.3643 | 0.316193 |
| Sfrp4 | Secreted frizzled-related protein 4 | Mm.42095 | NM_016687 | 1.3934 | 0.418827 |
| Wnt7a | Wingless-related MMTV integration site 7A | Mm.56964 | NM_009527 | 1.4446 | 0.689115 |
| Sfrp2 | Secreted frizzled-related protein 2 | Mm.19155 | NM_009144 | 1.5308 | 0.195642 |
| Wnt1 | Wingless-related MMTV integration site 1 | Mm.1123 | NM_021279 | 1.7136 | 0.76965 |
| Fgf4 | Fibroblast growth factor 4 | Mm.4956 | NM_010202 | 1.7666 | 0.256419 |
| Wnt2 | Wingless-related MMTV integration site 2 | Mm.33653 | NM_023653 | 1.867 | 0.229478 |
| Frzb | Frizzled-related protein | Mm.427436 | NM_011356 | 1.9308 | 0.131841 |
| Wnt8a | Wingless-related MMTV integration site 8A | Mm.558 | NM_009290 | 2.0137 | 0.207409 |
| Wisp1 | WNT1 inducible signaling pathway protein 1 | Mm.10222 | NM_018865 | 2.0305 | 0.235698 |
| Wnt16 | Wingless-related MMTV integration site 16 | Mm.137403 | NM_053116 | 2.079 | 0.144029 |

***Sorted by gene name***

| **Gene name** | **Description** | **Unigene** | **Refseq** | **Fold**  **Regulation** | **p-value** |
| --- | --- | --- | --- | --- | --- |
| Aes | Amino-terminal enhancer of split | Mm.180013 | NM_010347 | 1.0988 | 0.496949 |
| Apc | Adenomatosis polyposis coli | Mm.384171 | NM_007462 | -1.3731 | 0.189294 |
| Axin1 | Axin 1 | Mm.23684 | NM_009733 | -1.2142 | 0.25539 |
| Axin2 | Axin2 | Mm.71710 | NM_015732 | -1.4147 | 0.214857 |
| Bcl9 | B-cell CLL/lymphoma 9 | Mm.226175 | NM_029933 | -1.2022 | 0.009513 |
| Btrc | Beta-transducin repeat containing protein | Mm.119717 | NM_009771 | -1.6029 | 0.912582 |
| Ccnd1 | Cyclin D1 | Mm.273049 | NM_007631 | 1.2258 | 0.285213 |
| Ccnd2 | Cyclin D2 | Mm.333406 | NM_009829 | -1.3516 | 0.324436 |
| Csnk1a1 | Casein kinase 1, alpha 1 | Mm.26908 | NM_146087 | -1.2452 | 0.059202 |
| Csnk2a1 | Casein kinase 2, alpha 1 polypeptide | Mm.23692 | NM_007788 | -1.232 | 0.769848 |
| Ctbp1 | C-terminal binding protein 1 | Mm.240076 | NM_013502 | -1.0248 | 0.764843 |
| Ctnnb1 | Catenin (cadherin associated protein), beta 1 | Mm.291928 | NM_007614 | -5.8606 | 0.039424 |
| Ctnnbip1 | Catenin beta interacting protein 1 | Mm.299735 | NM_023465 | -1.5501 | 0.318734 |
| Daam1 | Dishevelled associated activator of morphogenesis 1 | Mm.474935 | NM_172464 | -1.7084 | 0.529445 |
| Dab2 | Disabled homolog 2 (Drosophila) | Mm.240830 | NM_023118 | 1.3643 | 0.316193 |
| Dixdc1 | DIX domain containing 1 | Mm.82598 | NM_178118 | -1.1164 | 0.907455 |
| Dkk1 | Dickkopf homolog 1 (Xenopus laevis) | Mm.214717 | NM_010051 | -1.7203 | 0.970153 |
| Dkk3 | Dickkopf homolog 3 (Xenopus laevis) | Mm.55143 | NM_015814 | -1.3741 | 0.751949 |
| Dvl1 | Dishevelled, dsh homolog 1 (Drosophila) | Mm.3400 | NM_010091 | -1.1331 | 0.484771 |
| Dvl2 | Dishevelled 2, dsh homolog (Drosophila) | Mm.5114 | NM_007888 | -1.1803 | 0.730529 |
| Ep300 | E1A binding protein p300 | Mm.258397 | NM_177821 | -2.1073 | 0.85848 |
| Fbxw11 | F-box and WD-40 domain protein 11 | Mm.28017 | NM_134015 | 1.0532 | 0.581796 |
| Fbxw4 | F-box and WD-40 domain protein 4 | Mm.254739 | NM_013907 | 1.1808 | 0.138696 |
| Fgf4 | Fibroblast growth factor 4 | Mm.4956 | NM_010202 | 1.7666 | 0.256419 |
| Fosl1 | Fos-like antigen 1 | Mm.6215 | NM_010235 | -1.4914 | 0.33896 |
| Foxn1 | Forkhead box N1 | Mm.4496 | NM_008238 | -1.4166 | 0.037657 |
| Frat1 | Frequently rearranged in advanced T-cell lymphomas | Mm.4573 | NM_008043 | -1.0382 | 0.274603 |
| Frzb | Frizzled-related protein | Mm.427436 | NM_011356 | 1.9308 | 0.131841 |
| Fzd1 | Frizzled homolog 1 (Drosophila) | Mm.246003 | NM_021457 | -3.192 | 0.43124 |
| Fzd2 | Frizzled homolog 2 (Drosophila) | Mm.36416 | NM_020510 | 1.0131 | 0.94147 |
| Fzd3 | Frizzled homolog 3 (Drosophila) | Mm.214687 | NM_021458 | 1.0007 | 0.968589 |
| Fzd4 | Frizzled homolog 4 (Drosophila) | Mm.86755 | NM_008055 | -1.7156 | 0.722114 |
| Fzd5 | Frizzled homolog 5 (Drosophila) | Mm.150813 | NM_022721 | -1.2618 | 0.545532 |
| Fzd6 | Frizzled homolog 6 (Drosophila) | Mm.4769 | NM_008056 | -2.2862 | 0.472544 |
| Fzd7 | Frizzled homolog 7 (Drosophila) | Mm.297906 | NM_008057 | 1.2232 | 0.155797 |
| Fzd8 | Frizzled homolog 8 (Drosophila) | Mm.184289 | NM_008058 | -2.6042 | 0.934413 |
| Fzd9 | Frizzled homolog 9 (Drosophila) | Mm.6256 | NM_010246 | -1.4482 | 0.650101 |
| Gsk3b | Glycogen synthase kinase 3 beta | Mm.394930 | NM_019827 | 1.008 | 0.920974 |
| Jun | Jun oncogene | Mm.275071 | NM_010591 | -1.4966 | 0.890874 |
| Kremen1 | Kringle containing transmembrane protein 1 | Mm.209989 | NM_032396 | 1.015 | 0.736121 |
| Lef1 | Lymphoid enhancer binding factor 1 | Mm.255219 | NM_010703 | -2.3653 | 0.039284 |
| Lrp5 | Low density lipoprotein receptor-related protein 5 | Mm.274581 | NM_008513 | -1.1865 | 0.404926 |
| Lrp6 | Low density lipoprotein receptor-related protein 6 | Mm.321990 | NM_008514 | -1.5205 | 0.604974 |
| Mapk8 | Mitogen-activated protein kinase 8 | Mm.21495 | NM_016700 | 1.1753 | 0.168594 |
| Mmp7 | Matrix metallopeptidase 7 | Mm.4825 | NM_010810 | -1.7537 | 0.092557 |
| Myc | Myelocytomatosis oncogene | Mm.2444 | NM_010849 | -1.4446 | 0.593277 |
| Nfatc1 | Nuclear factor of activated T-cells, cytoplasmic, calcineurin-dependent 1 | Mm.329560 | NM_016791 | 1.0463 | 0.325824 |
| Nkd1 | Naked cuticle 1 homolog (Drosophila) | Mm.30219 | NM_027280 | -1.7352 | 0.014712 |
| Nlk | Nemo like kinase | Mm.9001 | NM_008702 | -1.3141 | 0.005784 |
| Pitx2 | Paired-like homeodomain transcription factor 2 | Mm.246804 | NM_011098 | -1.1387 | 0.881615 |
| Porcn | Porcupine homolog (Drosophila) | Mm.443425 | NM_023638 | 1.291 | 0.315221 |
| Ppard | Peroxisome proliferator activator receptor delta | Mm.328914 | NM_011145 | -1.2393 | 0.101587 |
| Prickle1 | Prickle homolog 1 (Drosophila) | Mm.150314 | NM_001033217 | -1.01 | 0.619611 |
| Pygo1 | Pygopus 1 | Mm.273605 | NM_028116 | 1.1384 | 0.480802 |
| Rhoa | Ras homolog gene family, member A | Mm.757 | NM_016802 | -1.7267 | 0.70549 |
| Rhou | Ras homolog gene family, member U | Mm.168257 | NM_133955 | -2.2906 | 0.920277 |
| Ruvbl1 | RuvB-like protein 1 | Mm.42195 | NM_019685 | -1.1218 | 0.301791 |
| Sfrp1 | Secreted frizzled-related protein 1 | Mm.281691 | NM_013834 | -1.5016 | 0.66985 |
| Sfrp2 | Secreted frizzled-related protein 2 | Mm.19155 | NM_009144 | 1.5308 | 0.195642 |
| Sfrp4 | Secreted frizzled-related protein 4 | Mm.42095 | NM_016687 | 1.3934 | 0.418827 |
| Sox17 | SRY-box containing gene 17 | Mm.279103 | NM_011441 | -1.3057 | 0.519571 |
| Tcf7 | Transcription factor 7, T-cell specific | Mm.31630 | NM_009331 | -2.0596 | 0.000314 |
| Tcf7l1 | Transcription factor 7-like 1 (T-cell specific, HMG box) | Mm.440067 | NM_009332 | -1.5414 | 0.656847 |
| Tle1 | Transducin-like enhancer of split 1, homolog of Drosophila E(spl) | Mm.278444 | NM_011599 | 1.1313 | 0.158722 |
| Vangl2 | Vang-like 2 (van gogh, Drosophila) | Mm.36148 | NM_033509 | -2.1611 | 0.22507 |
| Wif1 | Wnt inhibitory factor 1 | Mm.32831 | NM_011915 | -2.1939 | 0.067763 |
| Wisp1 | WNT1 inducible signaling pathway protein 1 | Mm.10222 | NM_018865 | 2.0305 | 0.235698 |
| Wnt1 | Wingless-related MMTV integration site 1 | Mm.1123 | NM_021279 | 1.7136 | 0.76965 |
| Wnt10a | Wingless related MMTV integration site 10a | Mm.5130 | NM_009518 | -1.9184 | 0.537276 |
| Wnt11 | Wingless-related MMTV integration site 11 | Mm.22182 | NM_009519 | -1.1862 | 0.382355 |
| Wnt16 | Wingless-related MMTV integration site 16 | Mm.137403 | NM_053116 | 2.079 | 0.144029 |
| Wnt2 | Wingless-related MMTV integration site 2 | Mm.33653 | NM_023653 | 1.867 | 0.229478 |
| Wnt2b | Wingless related MMTV integration site 2b | Mm.10740 | NM_009520 | 1.1742 | 0.492405 |
| Wnt3 | Wingless-related MMTV integration site 3 | Mm.159091 | NM_009521 | -109.1163 | 0.000173 |
| Wnt3a | Wingless-related MMTV integration site 3A | Mm.1367 | NM_009522 | -1.174 | 0.522624 |
| Wnt4 | Wingless-related MMTV integration site 4 | Mm.20355 | NM_009523 | -1.0508 | 0.978068 |
| Wnt5a | Wingless-related MMTV integration site 5A | Mm.287544 | NM_009524 | -1.6002 | 0.056766 |
| Wnt5b | Wingless-related MMTV integration site 5B | Mm.321818 | NM_009525 | -1.2992 | 0.354011 |
| Wnt6 | Wingless-related MMTV integration site 6 | Mm.268282 | NM_009526 | 1.3246 | 0.405535 |
| Wnt7a | Wingless-related MMTV integration site 7A | Mm.56964 | NM_009527 | 1.4446 | 0.689115 |
| Wnt7b | Wingless-related MMTV integration site 7B | Mm.306946 | NM_009528 | -2.7135 | 0.798107 |
| Wnt8a | Wingless-related MMTV integration site 8A | Mm.558 | NM_009290 | 2.0137 | 0.207409 |
| Wnt8b | Wingless related MMTV integration site 8b | Mm.88365 | NM_011720 | -1.1596 | 0.701231 |
| Wnt9a | Wingless-type MMTV integration site 9A | Mm.218794 | NM_139298 | -2.7019 | 0.843866 |
